# IL-37 alleviates Coxsackievirus B3-induced viral myocarditis via inhibiting NLRP3 inflammasome-mediated pyroptosis

**DOI:** 10.1038/s41598-022-22617-y

**Published:** 2022-11-22

**Authors:** Lin Sun, Haitao Yuan, Gang Zhao

**Affiliations:** 1 Department of Cardiology, Shandong Provincial Hospital, Cheeloo College of Medicine, Shandong University, Jinan, Shandong 250021 China; 2 Department of Cardiology, Fuwai Hospital, National Center for Cardiovascular Disease, Chinese Academy of Medical Sciences and Peking Union Medical College, Beijing, 100037 China; 3 Department of Cardiology, Shandong Provincial Hospital Affiliated to Shandong First Medical University, Jinan, Shandong 250021 China

**Keywords:** Inflammasome, Antimicrobial responses

## Abstract

Our study aims to verify the potential effects and underlying mechanisms of IL-37 in Coxsackievirus B3 (CVB3)-induced viral myocarditis (VMC). VMC model was established by intraperitoneal injection of CVB3 into 6-week-old male balb-c mice on day 0. Each mouse of the IL-37-control group and IL-37-VMC CVB3 groups was intraperitoneally injected with IL-37 on day 4 and day 7. The cardiac function was evaluated by transthoracic echocardiography including LVEF, LVFE, IVSs and IVSd. Myocardial injury was measured by Elisa for serum cTnI. The inflammation infiltration and fibrosis were evaluated by hematoxylin and eosin (HE) staining and Masson staining. The expression levels of NLRP3 inflammasome components in pyroptosis were determined by western blot, Elisa, and immunofluorescent analysis. We also detected the expression of IL-37-IL-1R8 in PBMCs by immunofluorescence after injection with CVB3 and IL-37. Compared with the VMC group, mice received CVB3 and IL-37 have improved cardiac function, reduced inflammation infiltration and fibrosis, and with lower expression of cTnI, IL-1β, IL-18 and NLRP3 inflammasome component. IL-37 weakened the upregulation of GSDMD and phosphorylation of NF-κB p65 induced by CVB3. Exogenous addition of IL-37 with CVB3 further increases the production of IL-37-IL-1R8 -IL-18RA complex in vitro. Our findings indicate that IL-37 alleviates CVB3-induced VMC, which may be produced by inhibiting NLRP3 inflammasome-mediated pyroptosis, NF-κB signaling pathway, and IL-37-IL-1R8 -IL-18RA complex.

## Introduction

Viral myocarditis is a very important cause of heart failure and sudden death in young adults^1^. Although VMC is self-cure, some patients still developed dilated cardiomyopathy, malignant arrhythmia and heart failure^2^. VMC is an immune-related disease, mainly caused by infection of CVB3, HSV-6, PVB19, and et al.^3,4^. Many studies have been carried out to explore the pathogenesis of VMC, including direct injury caused by various viruses and cardiomyocyte damage mediated by overactive inflammation. However, the underlying pathogenesis of VMC is still unclear. The immune response is a double-edged sword^5^. On the one hand, the immune response to viral infection can promote virus clearance, but an over-activated immune response can cause a large number of inflammatory factors to be produced, which is called a "cytokine storm". These large amounts of cytokines can cause serious tissue damage, such as pyroptosis of myocardial cells and fibrosis of myocardial tissue^3,6^.

Pyroptosis is an inflammatory form of programmed cell death mediated by the inflammasome, activated by signal 1 and signal 2^7^. And pyroptosis has been confirmed as an important form of cardiomyocyte death in animal models of viral myocarditis. The inflammasome is an intracellular multiprotein complex consisting of a receptor protein, the adaptor protein and procaspase 1. NOD-like receptor family is the most studied pattern recognition receptor protein, including NLRP3. The transcription of NLRP3 inflammasomes is regulated by p65-NF-κB in signal 1. In signal 2, NLRP3 inflammasome is activated by pathogenic dsDNA, LPS, ROS, and other toxins^8^. Activated NLRP3 recruits the adaptor protein ASC (an apoptosis-associated speck-like protein containing a CARD), then recruits and activates caspase 1. Cleaved caspase 1 cleaves the precursor of IL-1β, IL-18, and GSDMD, leading to the release of proinflammatory cytokine IL-1β and IL-18. Gasdermin D is cleaved to form a peptide containing a nitrogen-terminal active domain, which binds to the cell membrane and induces cell membrane perforation, which eventually leads to cell lysis^7,9^. NLRP3 inflammasome-induced pyroptosis has an important role in the pathogenesis of VMC, and blocking it has been confirmed to be a potentially effective therapy^10^.

IL-37 is a novel member of the IL-1 cytokine family and is identified as an anti-inflammatory cytokine^11^. Intracellular IL-37 is activated by caspase 1 to reduce inflammation. While extracellularly IL-37 binds to IL-18Ra and IL-1R8 to suppress transduction of signal pathway^12^. It has been proved that IL-37 has therapeutic effects on tumors, colitis, and rheumatoid arthritis^13–15^. Especially in recent years, extracellular IL-37 has been confirmed to exert a regulatory role in autoimmune disease by inhibiting the activation of NLRP3 inflammasome^16,17^. For example, recombinant human IL-37 ameliorates temporomandibular joint inflammation via inhibiting the expression of NLRP3 and IL-1β, which can be reversed by knockdown of IL-1R8^18^. As a member of the IL-1 receptor family, IL-1R8 combines with IL-37 and IL-18RA to form a complex (IL-37-IL-1R8-IL-18RA complex), which negatively regulates the intensity of inflammation^8,19^.

We have previously proved that recombinant human IL-37 alleviates CVB3-induced viral myocarditis^20^. IL-37 may reduce inflammation by regulating the balance between Th17 and Treg cells. However, in viral myocarditis, the effects of IL-37 on NLRP3, pyrolysis, IL-1R8, etc. remain unclear. In this study, we found that IL-37 significantly ameliorated the signs of myocarditis and existed as a suppressor of NLRP3 inflammasome in a mouse VMC model. Moreover, we investigated the mechanisms of IL-37 via NF-κB pathway. Our current research aims to further explore the mechanism by which IL-37 affects VMC, so as to provide a new therapeutic option for the treatment of CVB3-induced myocarditis.

## Materials and methods

### Virus and mice

CVB3 (Nancy strain) was donated by the academy of Shandong provincial medical science and amplified in Hela cells. The virus was preserved at -80℃ in the academy of Shandong provincial medical science. The titer of CVB3 was measured by the median tissue culture infective dose (TCID50) assay as described previously^20^.

All animal experiments were approved by the Ethical Committee of Shandong University with the serial number (SDU.SPHEARC-A 057-2020) and performed strictly according to the ARRIVE guidelines. US National Institutes of Health Guidelines for the Use and Care of Laboratory Animals (NI Publications No. 85-23, revised 1985). The male 6-week-old balb-c mice were purchased from Beijing Vital River Laboratory Animal Technology Co., Ltd (Beijing, China), which were bred in a standard specific pathogen-free (SPF) environment in the academy of Shandong Provincial Medical Science^20^. We randomly assigned 40 mice into 4 groups: control group (n = 8), IL-37-injected mice control group (IL-37 control group, n = 8), CVB3-infected mice group (VMC group, n = 12), and CVB3-infected mice with IL-37-injected group (IL-37 VMC group, n = 12).

### VMC model establishment

VMC model was generated by intraperitoneal injection of 15 μl 10^9^ TICD50 CVB3 into 6-week-old male balb-c mice on day 0 as previously described^21–23^. The control group and IL-37-control group received intraperitoneal injection of equal volume of PBS. On day 4 and day 7, each mouse of the IL-37-control group and IL-37-VMC CVB3 groups was intraperitoneally injected with 2 mg IL-37 (diluted in 200 ml PBS) as previously described^20,24^. Mice in the control group and VMC group were intraperitoneally injected with 200 ml PBS. All mice were sacrificed on day 9 with serum and hearts collected.

### Cardiac function measurement

On day 9, we performed transthoracic echocardiography by the Vevo 2100 ultrasound imaging system (Visual Sonics, Toronto, Canada) with continuous inhalation of isoflurane^25^. The left ventricular ejection fraction (LVEF), left ventricular fractional shortening (LVFS), interventricular septum end-systolic thickness (IVSs) and interventricular septum end-systolic thickness (IVSd) was measured.

### Histological analysis

Mice were sacrificed on day 9 under anesthesia with pentobarbital. After perfusion with normal saline, hearts were fixed in 4% paraformaldehyde for 48 h, and undergone dehydration. Then hearts were embedded in paraffin and sectioned into 5-micron-thick sections for subsequent histology analysis. Sections were stained with HE and Masson staining. As described in previous studies^22^, the pathologic grading of inflammation infiltration including five scales: 0 = no inflammatory cell infiltration of the cross-sectional area; 1 = not more than 25% myocardium is involved with inflammatory foci; 2 = 25% to 50% myocardium is involved with inflammatory foci; 3 = 50%-75% of myocardium is involved with inflammatory foci; 4 = more than 75% myocardium is involved in inflammatory foci^26^. Furthermore, sections were incubated with the primary antibody specific for GSDMD (1:1000, Abcam, UK) overnight and incubated with fluorescent secondary antibody (1:100, Abbkine, China)^27^. Digital images were collected with Cellsens standard (Olympus, Japan). The levels of GSDMD were analyzed by Image-Pro Plus 6.0 software (MEDIA CYBERNETICS, USA).

### Detect the interaction between IL-37 and IL-1R8 in peripheral blood mononuclear cells (PBMCs) using immunofluorescence

PBMCs were isolated from the peripheral blood of healthy donors and separated by density gradient centrifugation at 1500 rpm for 20 min. All cells were cultured in RPMI 1640 medium (Gibco, USA) supplemented with 10% fetal bovine serum (Gibco, USA) in a 5% CO_2_ incubator at 37 °C. Cells were stimulated with IL-37 or CVB3 or CVB3 plus IL-37. We co-stained IL-37 and IL-18Rα in human peripheral mononuclear cells through immunofluorescence. Anti-IL-1R8 (Santa Cruz, USA), anti-IL-37 (eBioscience, USA), and mouse IgG (Cell Signaling Technology, USA) were used. Digital images were collected with Cellsens standard (Olympus, Japan). The expression level of IL-1R8 was analyzed by Image-Pro Plus 6.0 software (MEDIA CYBERNETICS, USA). All experimental procedures are performed as described previously^8,28^.

### Enzyme‑linked immunosorbent assay (ELISA)

The levels of cTnI, IL-1β and IL-18 in peripheral serum were measured with ELISAs (Elabscience, China) strictly following the instructions of the kit.

### Western blot analysis

Heart tissue was ground with liquid nitrogen and lysed using RIPA lysis buffer (Beyotime Biotechnology, China) containing protein phosphorylase inhibitor (Beyotime Biotechnology, China) and protease inhibitor cocktail (MedChemExpress, USA) on ice, and then undergone centrifugation at 12,000*g* for 10 min at 4 °C. The protein concentration of supernatants was determined by BCA assay (Beyotime Biotechnology, China). Protein samples were separated by SDS-PAGE electrophoresis and transferred to polyvinylidene fluoride (PVDF) membranes (Millipore, USA). All the blots were cut prior tp hybridization with antibodies. After blocking, membranes were incubated with primary antibodies against NLRP3 (1:1000, Abcam, UK), ASC (1:1000, Abcam, UK), cleaved caspase-1 (1:1000, Cell Signaling Technology, USA), NF-κB p65 (1:1000, Cell Signaling Technology, USA), and NF-κB phosphorylation of p65 (1:1000, Cell Signaling Technology, USA), GADPH (1:5000, Proteintech, China), and β-actin (1:3000, Proteintech, China) overnight at 4 °C. Membranes were incubated with anti-rabbit IgG (1:5000, Proteintech, China) or anti-goat IgG (1:5000, Proteintech, China) for 1 h at room temperature. Bands were visualized using ECL solution (Millipore, USA). The levels of target molecules were analyzed by densitometry using ImageJ software (National Institutes of Health, USA).

### Real time PCR analysis

The total RNA was extracted from frozen heart tissues using Trizol reagent (Invitrogen, USA). A total of 400 ng of RNA per sample was used for reverse transcription and PCR assays (Takara, Japan). The specific primers for NF-kB p65 and β-actin were as follows: murine β-actin: 5′-TGTTACCAACTGGGACGACA-3′, 5′-CTGGGTCATCTTTTCA CGGT-3′; murine NF-κB p65: 5′-CTGCCGAGTAAACCGGAACT-3′, 5′-GCCTGGTCCCGTGAAATACA-3′. The expression levels of NF-κB gene were normalized to that of β-actin, which served as the endogenous control. Relative quantification of gene expression was performed via the 2^−ΔΔCT^ method.

### Statistical analysis

All the statistical analyses were performed using the GraphPad Prism (version 6.0). All data were expressed as the mean ± S.E.M and all experiments were performed at least three independent times. Comparisons between groups were performed by one-way ANOVA with post hoc analyses performed using the Student–Newman–Keuls method. The differences between 2 groups were compared by Student's unpaired t-test. *P* < 0.05 was considered statistically significant.

### Ethics approval and consent to participate

All animal experiments were approved by the Ethical Committee of Shandong University with the serial number (SDU.SPHEARC-A 057-2020) and performed strictly according to US National Institutes of Health Guidelines for the Use and Care of Laboratory Animals (NI Publications No. 85-23, revised 1985).

## Results

### CVB-3 induced mouse viral myocarditis model was successfully constructed

**Figure 1:**
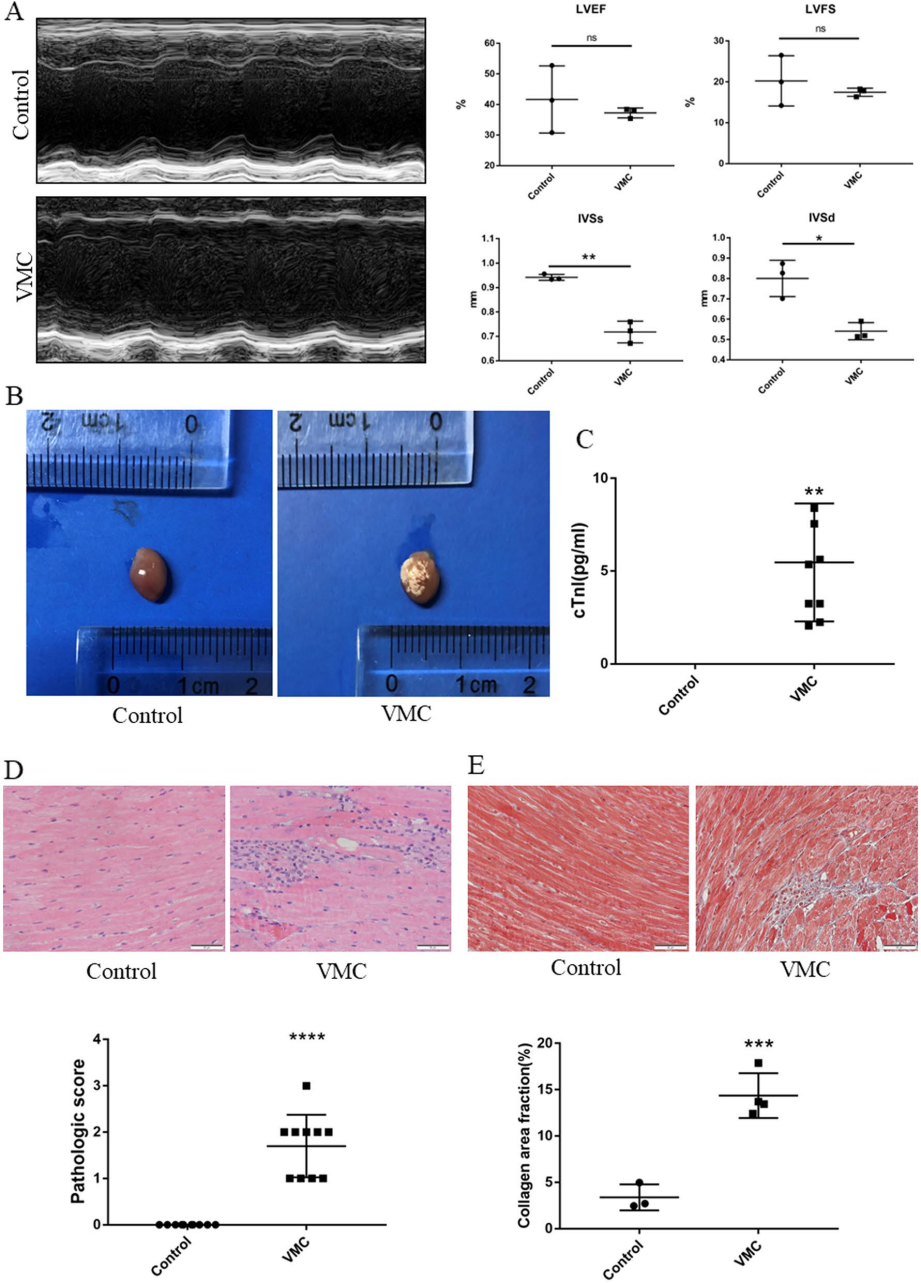
Establishment of the VMC model. (**A**) M-mode echocardiographic images of mice in control group and VMC group and the LVEF, LVFS, IVSs, IVSd from the echocardiographic data. (**B**) Hearts of mice in control group and VMC group. (**C**) The levels of cTnI in serum of mice in control group and VMC group were measured by ELISA. (**D**, **E**) Histopathological changes in heart tissue of control group and VMC group were examined by H&E staining and Masson staining. The pathologic score of mice in control group and VMC group. (*P < 0.01, **P < 0.001, ****P < 0.00001, ns: not significant.)

First, we established the VMC model by intraperitoneal injection of 15 μl 10^9^ CVB3 into 6-week-old male balb-c mice. Before mice were sacrificed on day 9, transthoracic echocardiography was performed. Compared with the control group, LVEF, LVFS was decreased in VMC group, but there was no significant difference (both *P* > 0.05). IVSs (*P* < 0.01) and IVSd (*P* < 0.05) were also decreased in the VMC group (Fig. 1A). It can be seen from the anatomical pictures of the heart that there are many patch-like lesions on the surface of the heart of the mice in the VCM group, but there is no patch-like pathological change on the surface of the heart of the control group (Fig. 1B). We also confirmed that the serum cTnI of the VMC group was significantly higher than that of the control group (*P* < 0.001) (Fig. 1C). HE staining showed apparent inflammatory infiltration and higher pathological scores in the VMC group than that in the control group (*P* < 0.00001) (Fig. 1D). Moreover, Masson staining showed more collagen deposition in the hearts of VMC mice compared with the control group (Fig. 1E). All mentioned data above indicate a successful establishment of the VMC model.

### CVB3 infection up-regulated the levels of IL-1β and IL-18 in serum and GSDMD in heart tissue

**Figure 2:**
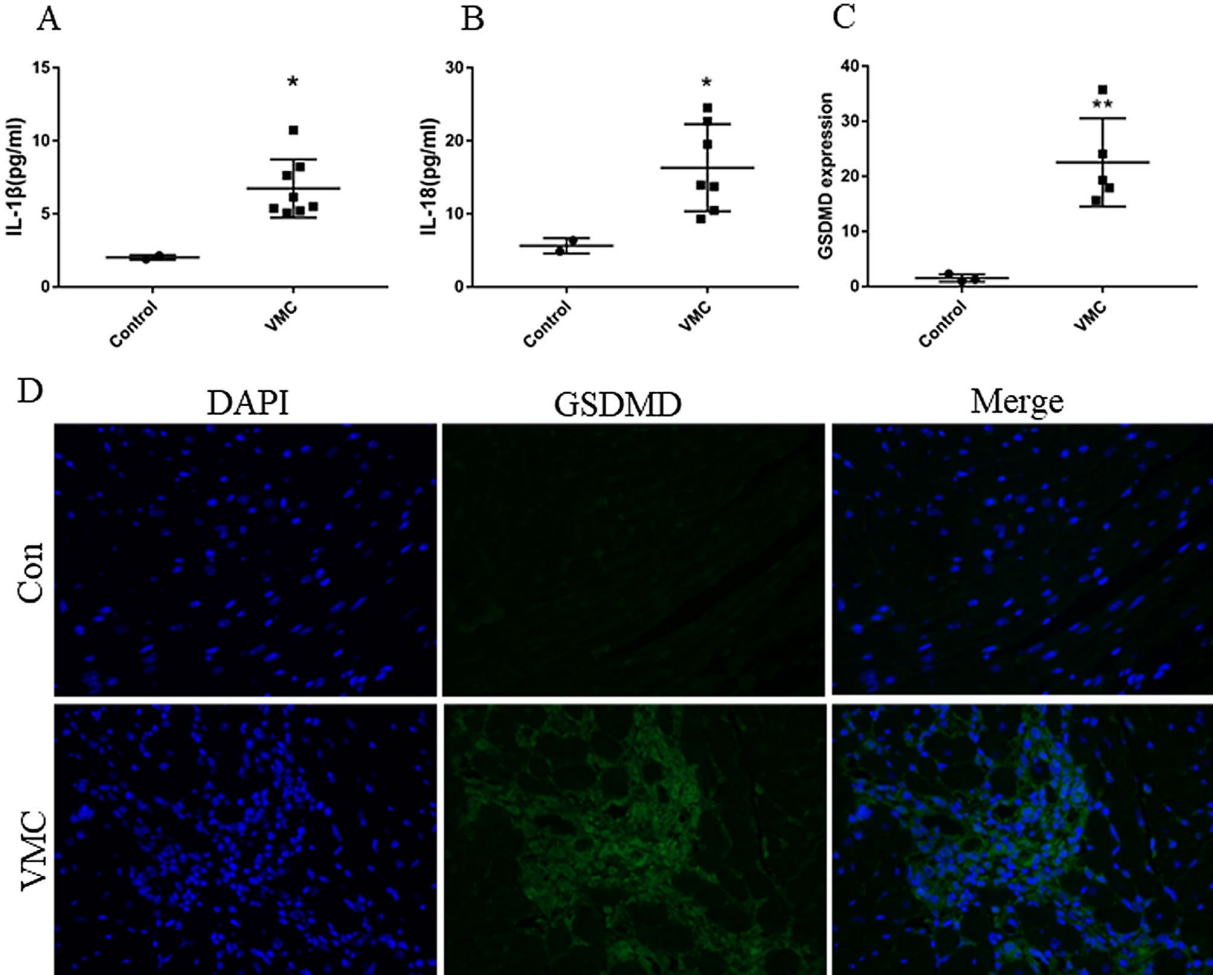
Pyroptosis is overactivated in the VMC group. (**A**, **B**) The levels of IL-18 and IL-1β in serum of mice in control group and VMC group were measured by ELISA. (**C**) Analysis of the expression of GSDMD in heart tissue from the immunofluorescent data. (**D**) The representative pictures of immunofluorescent analysis of GSDMD expression. (*P < 0.01, **P < 0.001.)

We assayed the protein levels of IL-18, IL-1β and detected the expression of GSDMD. Compared with the control group, the levels of serum IL-18 and serum IL-1β were significantly increased in the VMC group (both *P* < 0.01) (Fig. 2A,B). The GSDMD protein was detected by immunofluorescence, which showed more GSDMD expression in the VMC group (*P* < 0.001) (Fig. 2C,D).

### IL-37 alleviates myocardial injury and impaired heart function in CVB3-induced VMC

**Figure 3:**
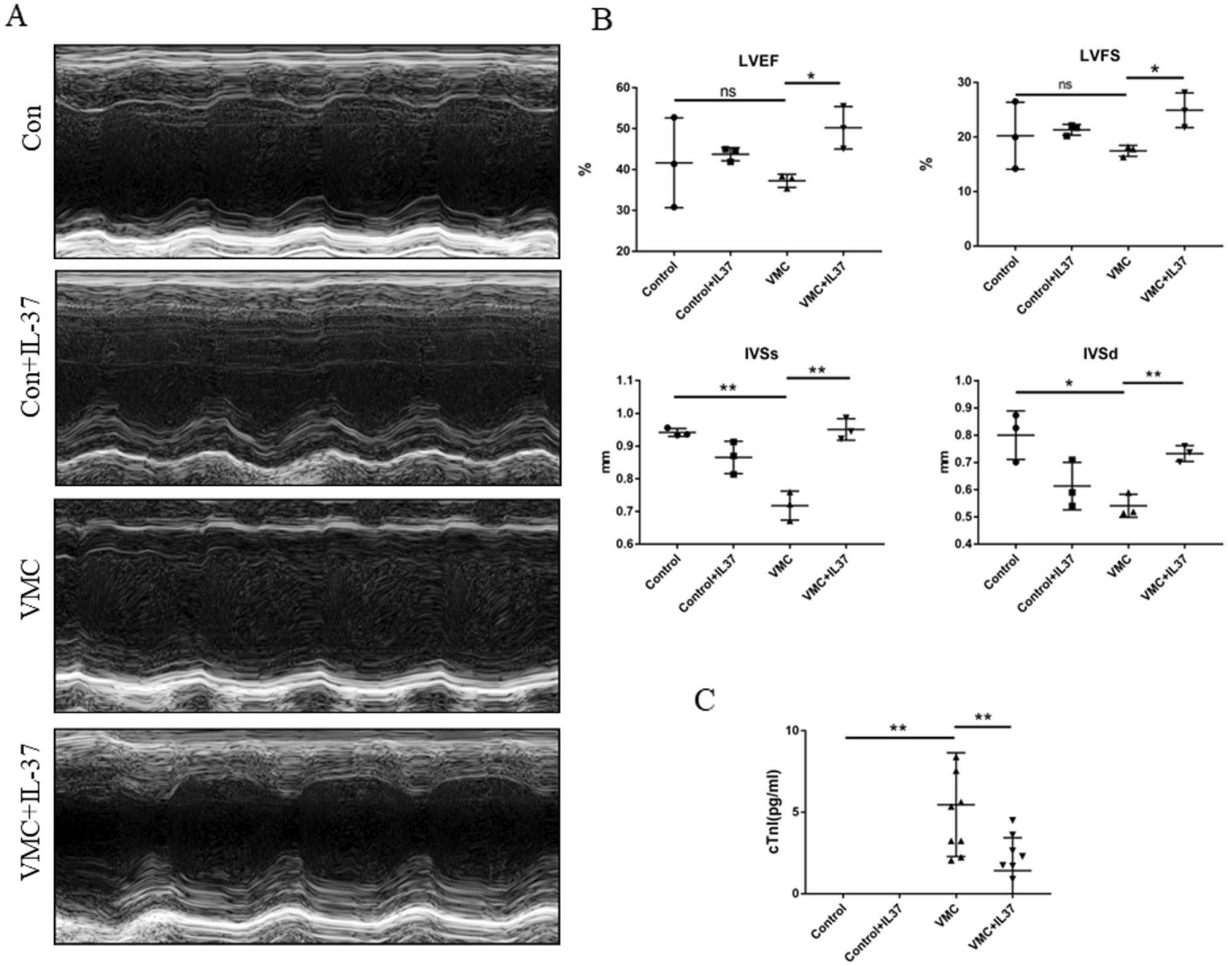
IL-37 alleviates myocarditis in CVB3-induced VMC mice. (**A**, **B**) M-mode echocardiographic images of mice in control group and VMC group and the LVEF, LVFS, IVSs, IVSd from the echocardiographic data. (**C**) The levels of cTnI in serum of mice in four groups were measured by ELISA. (Con: control group, *P < 0.01, **P < 0.001, ****P < 0.00001, ns: not significant.)

To investigate the role of IL-37 in VMC, we injected IL-37 on day 4 and day 7. Echocardiography showed that IL-37 treatment resulted in better cardiac function parameters in mice with viral myocarditis, including LVEF (*P* < 0.05), LVFS (*P* < 0.05), IVSs (*P* < 0.01) and IVSd (*P* < 0.01) (Fig. 3A,B). We further found that IL-37 treatment significantly attenuated the elevation of cTnI in serum induced by CVB3 infection (*P* < 0.001) (Fig. 3C). Taken together, the above results indicate IL-37 can alleviate myocardial damage induced by CVB3 infection.

### IL-37 attenuated CVB3-induced inflammatory cell infiltration and collagen tissue deposition in myocardial tissue

**Figure 4:**
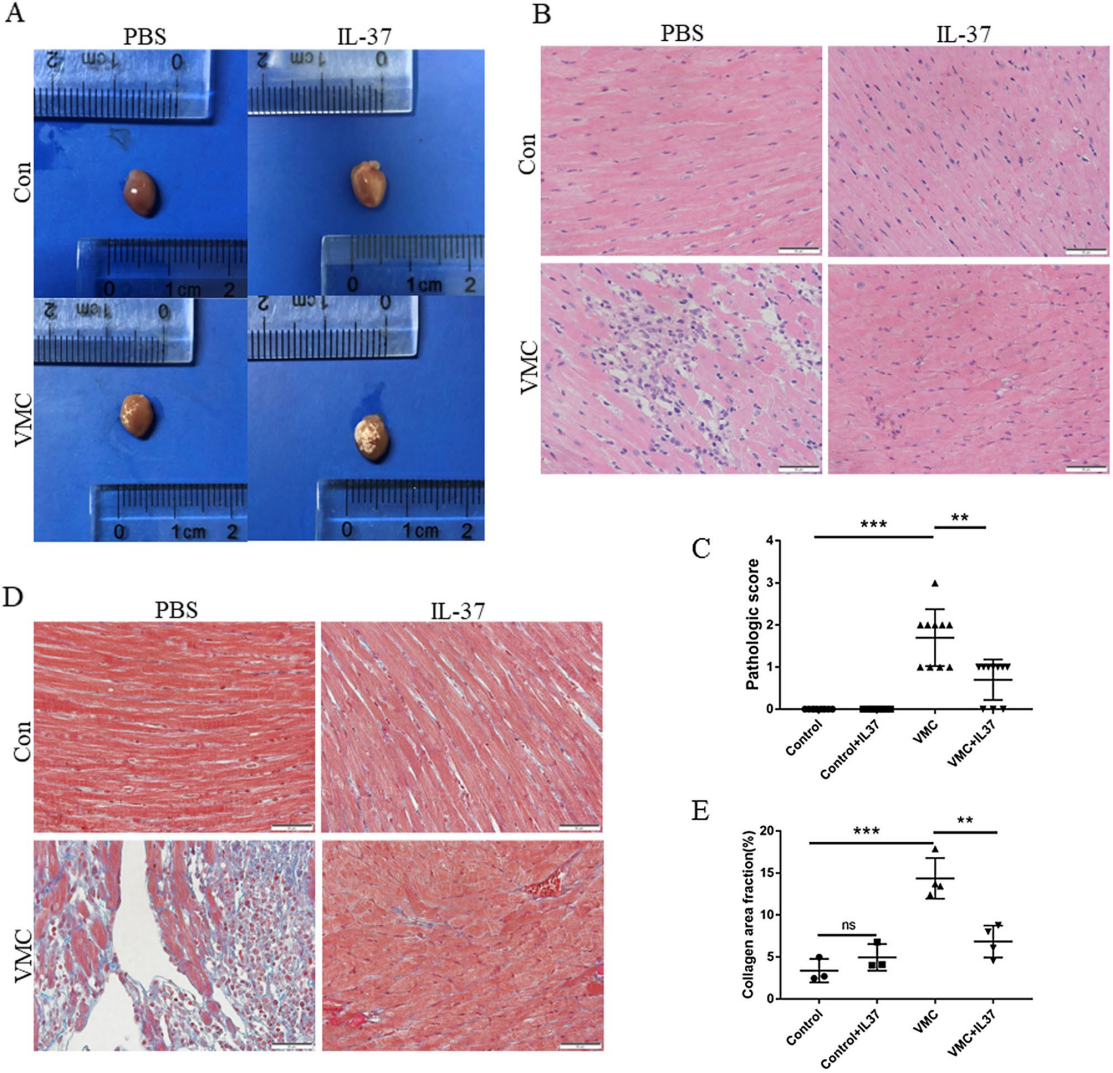
IL-37 attenuated inflammatory cell infiltration and collagen tissue deposition. (**A**) Hearts of mice in all groups. (**B**, **D**) Histopathological changes in heart tissue of four groups were examined by H&E staining and Masson staining. (**C**, **E**) The pathologic score of mice in control group and VMC group. (*P < 0.01, **P < 0.001, ****P < 0.00001, ns: not significant.)

It can be seen from the anatomical pictures of the heart that there are many patch-like lesions on the surface of the heart of the mice in the VCM group, but IL-37 treatment obviously reduced the area of patch-like lesions (Fig. 4A). Using HE staining and Masson staining, we found that IL-37 treatment significantly reduced the inflammatory cell infiltration score (*P* < 0.001) (Fig. 4B,C) and fibrous tissue deposition in myocardial tissue induced by CVB3 infection (Fig. 4D,E).

### IL-37 attenuated the activation of NLRP3 inflammasome and the production of IL-1β and IL-18

**Figure 5:**
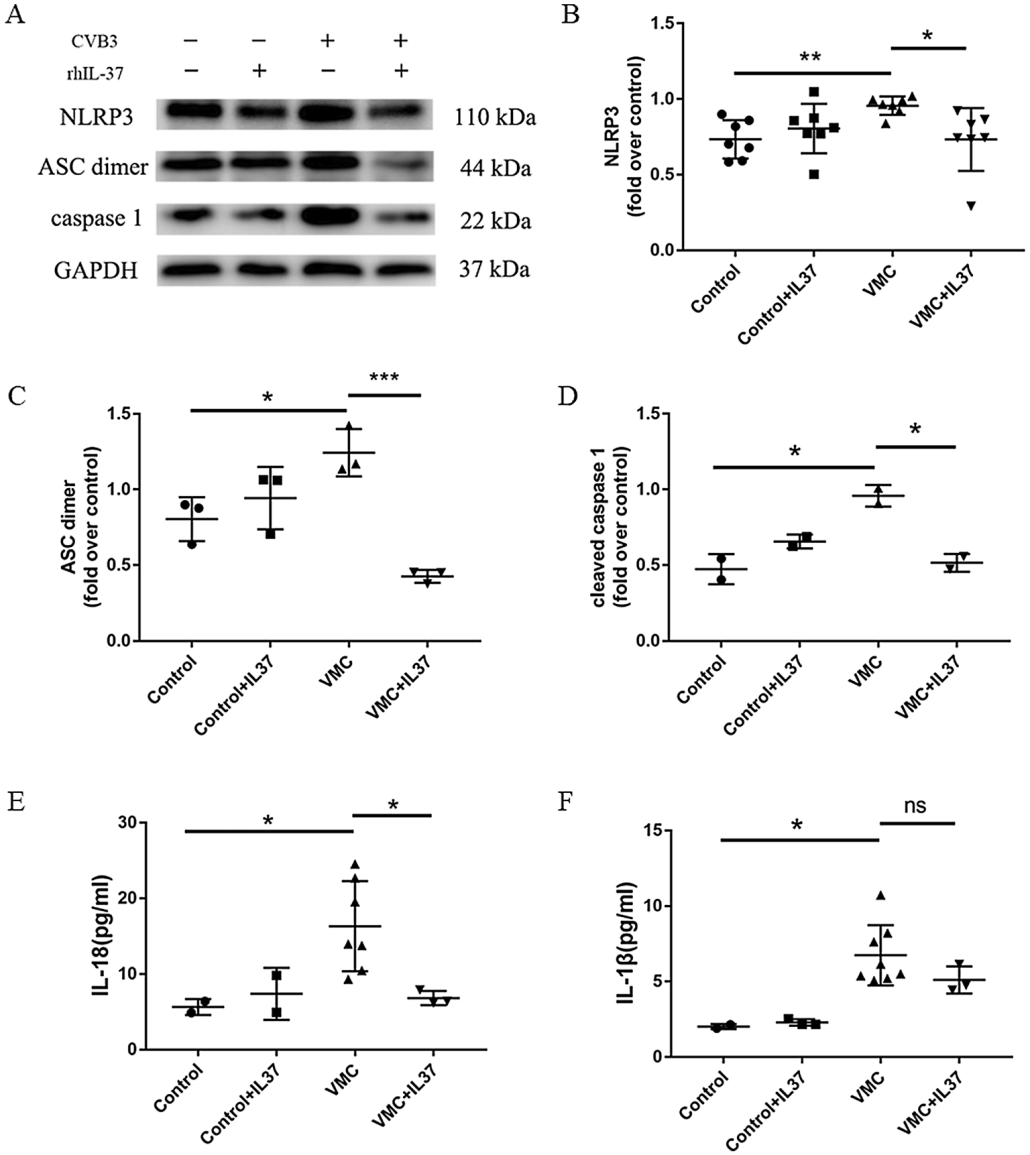
IL-37 attenuated the activation of NLRP3 inflammasome and the production of IL-1β and IL-18. (**A**) The expression of NLRP3, ASC dimer and cleaved caspase 1 in heart tissues were measured by western blot. (**B**–**D**) Bar graphs of quantitative analysis of NLRP3, ASC dimer and cleaved caspase 1. (**E**, **F**) The levels of IL-18 and IL-1β in serum of mice in all groups were measured by ELISA. (*P < 0.01, **P < 0.001, ***P < 0.0001, ns: not significant.)

In view of the strong association between IL-37 and NLRP3, we further investigated the effect of IL-37 on NLRP3 inflammasome in CVB3-induced myocarditis. The NLRP3 inflammasome components in heart tissue including NLRP3 (*P* < 0.01), ASC dimer (*P* < 0.0001) and cleaved caspase 1 (*P* < 0.01), were significantly higher in the VMC group compared to the control group (Fig. 5A–D). However, IL-37 treatment significantly attenuated these changes induced by CVB3. In addition, CVB3 infection increased the levels of IL-1β and IL-18 (*P* < 0.01) in the serum of mice, while IL-37 treatment apparently attenuated this change (Fig. 5E,F). It was worth mentioning that IL-1β in VMC + IL-37 group was significantly lower than that in VMC group, but there was no significant difference (*P* > 0.05) (Fig. 5F). The result indicated that IL-37 treatment attenuated the activation of NLRP3 inflammasomes induced by CVB3 infection.

**Figure 6:**
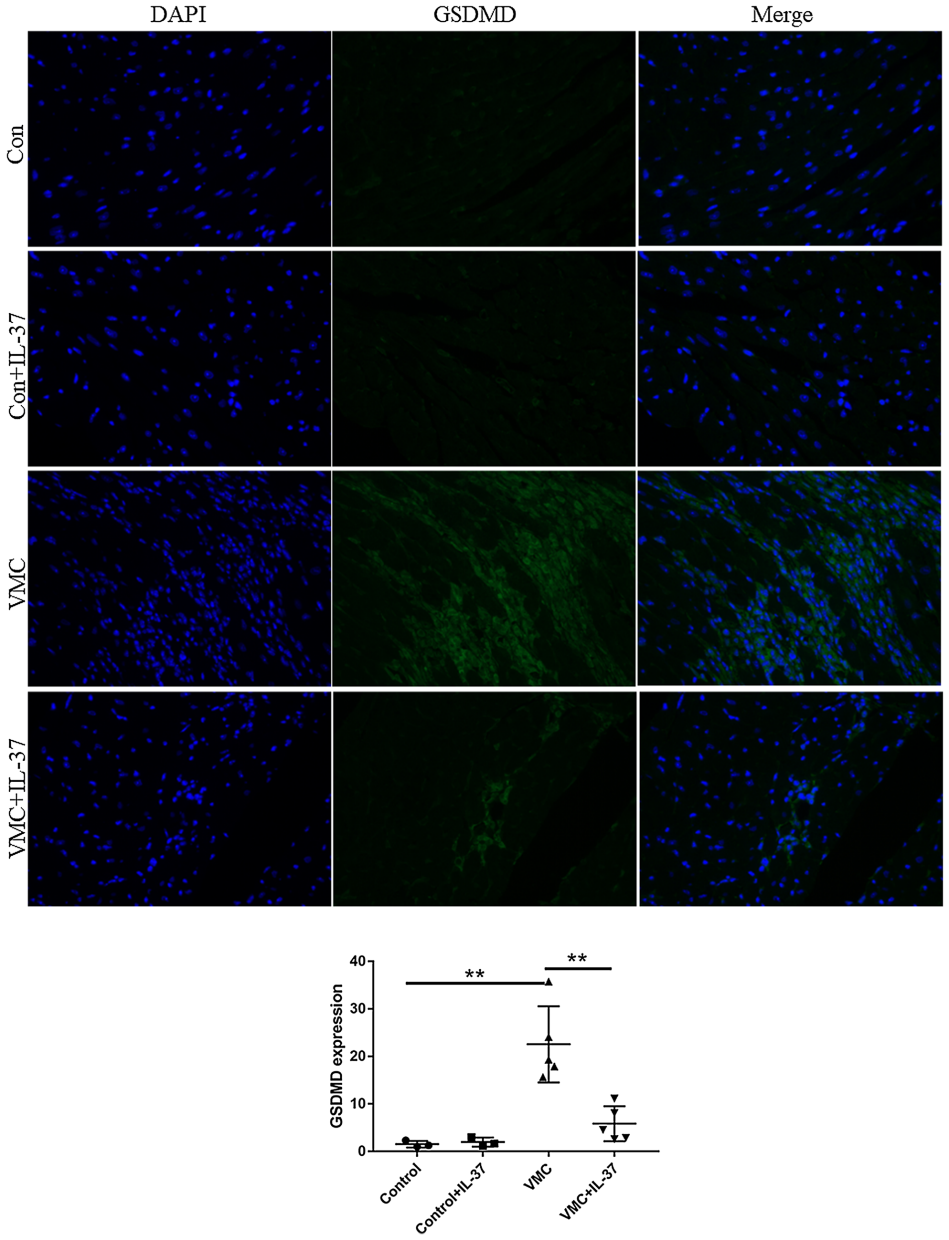
The representative pictures of immunofluorescent analysis of GSDMD expression in four groups.

Considering the effect of NLRP3 on cell pyrolysis and the importance of GSDMD protein on cell pyrolysis, we further tested GSDMD. We found in this study that the levels of GSDMD protein were markedly decreased in VMC with IL-37 treatment compared with the VMC group (*P* < 0.001), which implied that IL-37 inhibits pyroptosis in CVB3-induced myocarditis (Fig. 6).

### IL-37 weakens the NF-κB signaling pathway in the heart tissue of mice with VMC

**Figure 7:**
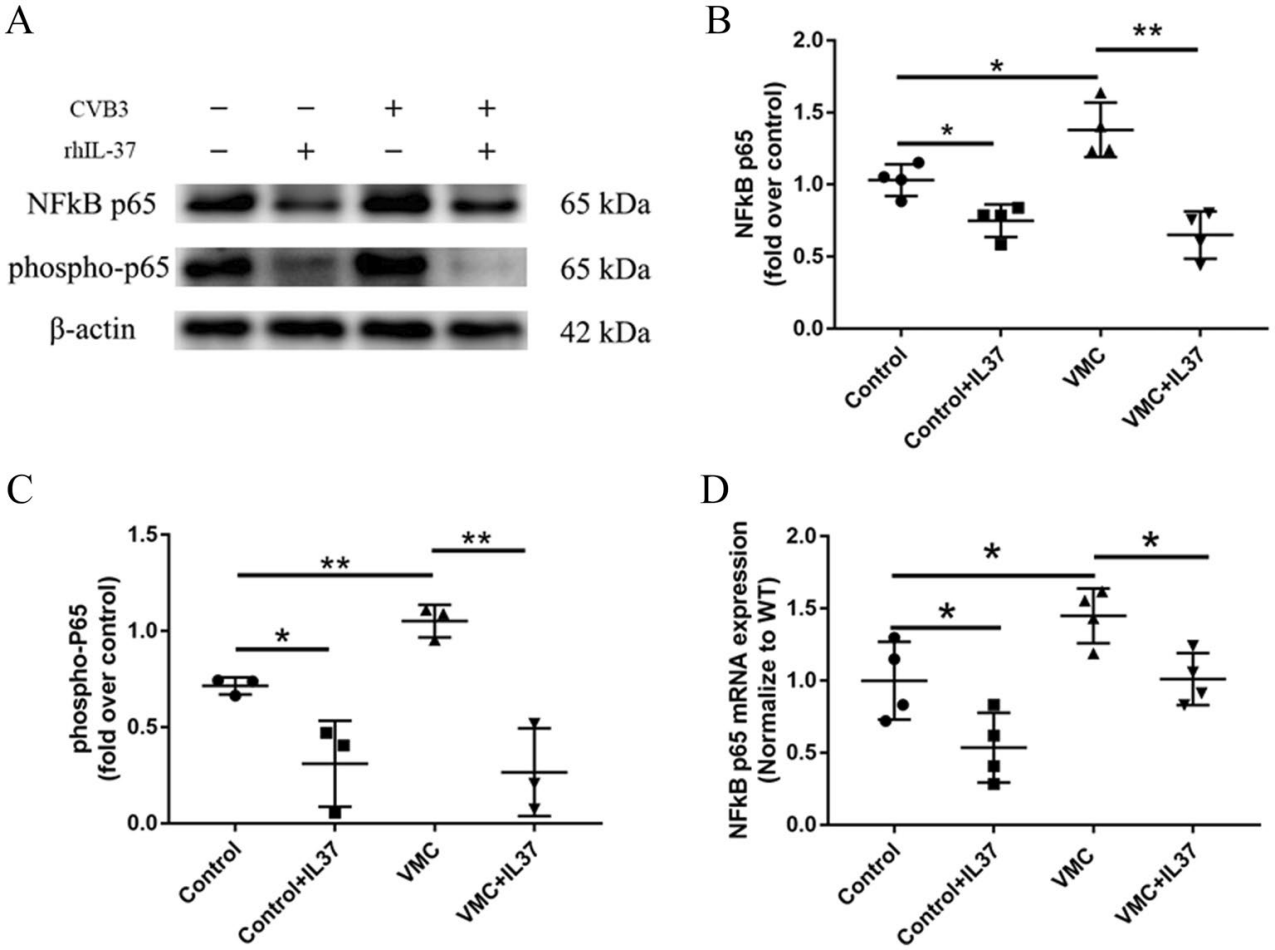
IL-37 may decrease pyroptosis via inhibiting the expression of NF-κB. (**A**) The representative pictures of NF-κB p65 and phosphorylation of p65 expression of in heart tissues assayed by western blot. (**B**, **C**) Bar graphs of quantitative analysis of NF-κB p65 and phosphorylation of p65 protein. (**D**) The mRNA levels of NF-κB were detected by real time PCR. (*P < 0.01, **P < 0.001).

To further investigate the potential mechanism of IL-37 in VMC, we also assayed the NF-κB signaling pathway by western blot and real time PCR. We found that compared with the control group, the protein levels of NF-κB p65 (*P* < 0.01) and the phosphorylation of p65 (*P* < 0.001) were significantly increased in the VMC group. Compared with the VMC group, IL-37 treatment significantly down-regulated the expression (*P* < 0.01) and phosphorylation level (*P* < 0.001) of NF-κB p65 (Fig. 7A–C). We further confirmed using real time PCR that the mRNA levels of NF-κB were markedly upregulated in the VMC group. However, IL-37 treatment significantly attenuated the increase and phosphorylation of NF-κB induced by CVB3 (all *P* < 0.01) (Fig. 7D). All these results indicate that IL-37 inhibits NLRP3 expression via inhibiting the NF-κB signaling pathway.

### CVB3 infection slightly increased the combination of IL-37 and IL-1R8, and the exogenous supplementation of IL-37 further enhances the binding

**Figure 8:**
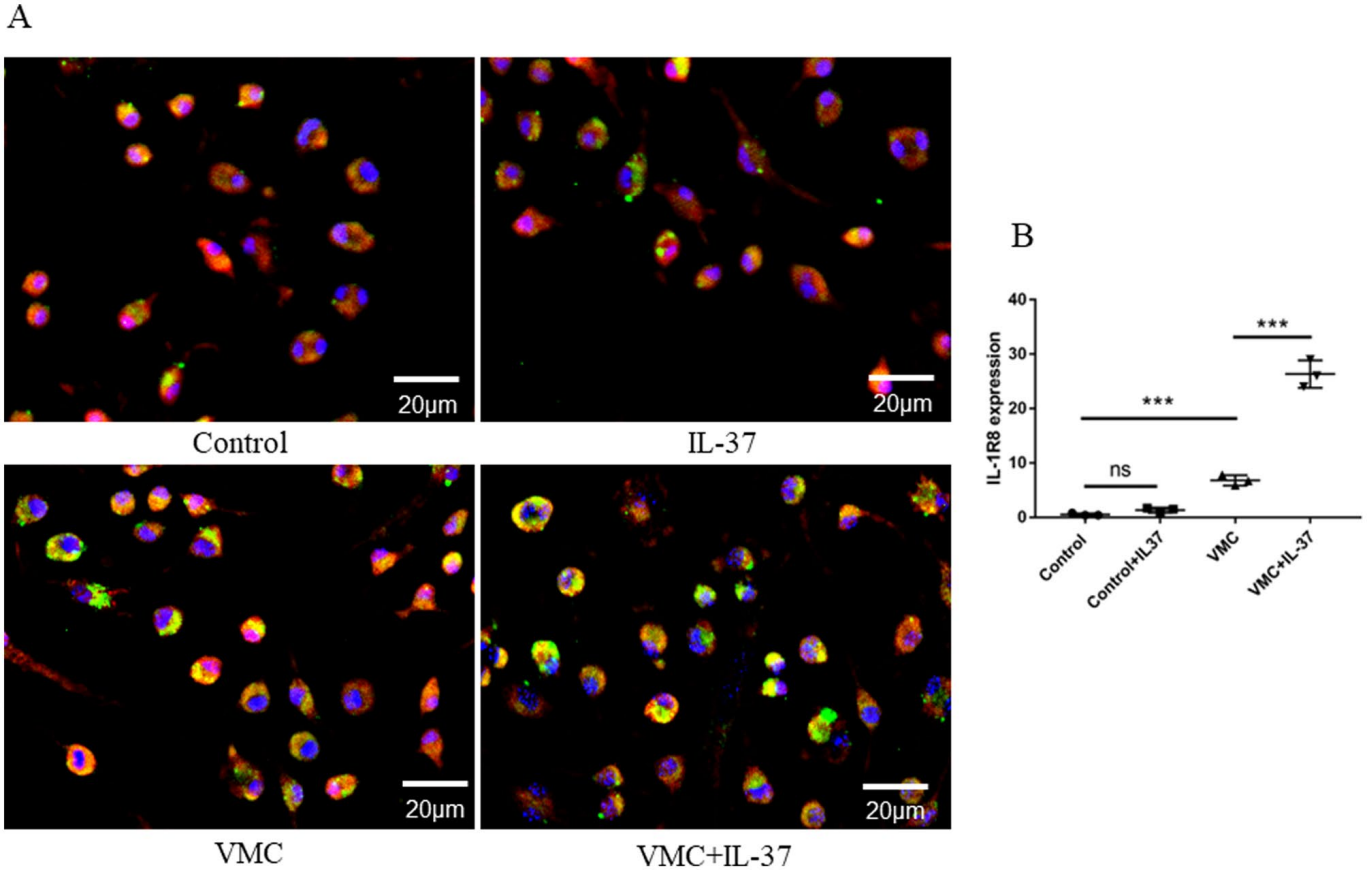
Binding of IL-37 to IL-1R8 in the PBMCs. Exogenous supplementation of IL-37 enhanced the combination of IL-37 and IL-1R8. Both injection of IL-37 and CVB3 virus increase the binding of IL-37-IL-1R8 complex respectively. CVB3 infection with exogenous IL-37 enhanced the mutual binding of IL-37 and IL-1R8 (P < 0.01 VS Control and IL-37 alone). Original blots are presented in Supplementary Figures.

It is reported that the formation of endogenous ligand receptor complex (IL-37-IL-1R8 -IL-18RA) is an important step for the anti-inflammatory activity of IL-37 upon exogenous stimulus. In this study, we found using immunofluorescence that exogenous IL-37 addition alone slightly increased the IL-37-IL-1R8 complex compared with control group, but the difference between the two was not statistically significant (Fig. 8). Compared with the control group and IL-37 alone group, CVB3 infection definitely enhanced the mutual binding of IL-37 and IL-1R8 (*P* < 0.01 VS Control and IL-37 alone). Based on the effect of CVB3, the addition of exogenous IL-37 further enhanced the IL-37-IL-1R8 complex formation (Fig. 8).

## Discussion

The current study has demonstrated that IL-37 treatment significantly reduces cardiac injury caused by CVB3 infection, as evidenced by improved cardiac function and decreased myocardial necrosis markers. We also confirmed that IL-37 significantly reduced CVB3-induced inflammatory cell infiltration and fibrous deposition in myocardial tissue. Furthermore, we confirmed that IL-37 significantly attenuates the activation of NLRP3 inflammasomes and the up-regulation of GSDMD caused by CVB3 infection. CVB3 infection up-regulates NF-κB p65 and enhances its phosphorylation, and slightly increases the interaction between IL-37 and IL-1R8. IL-37 treatment significantly weakened the activation of NF-κB signal caused by CVB3 infection, and further enhanced the interaction between IL-37 and IL-1R8. The protective effect of IL-37 on the heart against CVB3 infection may be related to its effect on the NLRP3 inflammasome-GSDMD, NF-κB p65 and IL-37-IL-1R8-IL-18RA complex.

IL-37, as a unique member of the IL-1 family of cytokines, functions as a natural suppressor of inflammatory and immune responses^8,17^. Transgenic mice expressing human IL-37 and mice treated with IL-37 are protected from many different experimental models of inflammation, including endotoxin shock, colitis, lung injury, arthritis and inflammation-induced arthritis^16,17^. In humans, some researchers found IL-37 levels are abnormal in patients with inflammation-related diseases^8^. However, whether IL-37 has an effect on viral myocarditis caused by CVB3 has not been reported yet. In the current study, we confirmed that IL-37 protects the heart against damage caused by CVB3. Not surprisingly, IL-37 inhibits the infiltration of inflammatory cells in myocardial tissue caused by CVB3. And IL-37 significantly inhibited the up-regulation of IL-1β and IL-18 in plasma caused by CVB3 infection. Our results suggest that IL-37 is likely to impose a restriction on excessive inflammation.

IL-1β and IL-18 are both active inflammatory mediators formed after the activated NLRP3 inflammasome cleaves their respective precursors^29,30^. Many studies have confirmed that NLRP3 inflammasome, which is a widely studied inflammasome, is related to infection, tumor, autoimmune diseases^29–31^. The activation of NLRP3 inflammasome is involved in the pathogenesis of many inflammatory diseases^32^. IL-37 is an anti-inflammatory factor in IL-1 family, which plays a role in lung cancer, rheumatoid arthritis and many other diseases^33,34^. Extracellular IL-37 has been confirmed to exert a regulatory role in autoimmune disease by inhibiting the activation of NLRP3 inflammasome^8,16,17^. And, IL-37 treatment ameliorates temporomandibular joint inflammation via inhibiting the expression of NLRP3 and IL-1β, which can be reversed by knockdown of IL-1R8^8^. Our previous studies has confirmed that recombinant human IL-37 alleviates CVB3-induced viral myocarditis by regulating the ratio of Th17/Treg cells^20^. In the current study, we found that NLRP3 inflammasome was highly expressed in viral myocarditis with increased levels of IL-18 and IL-1β. In CVB3-induced viral myocarditis, IL-37 treatment obviously weakened the upregulation of NLRP3 inflammasomes (NLRP3, ASC, and caspase-1), and the activation of NLRP3 inflammasomes as evidenced by decreased inflammatory mediators (IL-1β and IL-18). Therefore, IL-37's inhibition of NLRP3 inflammasome in VMC may be an important reason for its anti-inflammatory effect.

In viral myocarditis, myocardial cell pyroptosis is an important factor leading to the continuous decline of heart function and the activation of NLRP3 inflammasome is an important step leading to pyrolysis^8,10^. In this study, our findings indicate activated pyroptosis in viral myocarditis and we have confirmed that IL-37 significantly inhibits the activation of NLRP3 inflammasome caused by CVB3. Therefore, we speculated that IL-37 can improve viral myocarditis by inhibiting the activation of NLRP3 inflammasome-mediated pyroptosis. Therefore, in this study, we detected GSDMD, an indispensable target in the process of pyroptosis, and our results confirmed that IL-37 significantly inhibited the up-regulation of GSDMD caused by CVB3 infection. This probably means that IL-37 is likely to reduce the pyrolysis of cardiomyocytes by inhibiting the NLRP3 inflammasome.

NF-κB is the common pivotal target of multiple inflammatory signaling pathways and plays a key role in the expression of NLRP3^9,29,35^. Therefore, we detected in this study the expression of NF-κB p65 and phosphorylation of p65 and found that IL-37 can downregulate the expression of NF-κB p65 and phosphorylation of p65 protein. We further found that IL-37 can inhibit the mRNA expression of NF-κB p65. In viral myocarditis, IL-37's inhibition of NF-κB signaling pathway may be an important means to inhibit the expression of a variety of pro-inflammatory genes.

In addition, many researchers have confirmed that IL-37 needs to form a complex with IIL-1R8 and IL-18RA in a variety of inflammation-related disease models to exert its anti-inflammatory effects^19,36^. However, there is no relevant experimental data for viral myocarditis. In the current experiment, we found that IL-37 treatment alone can only slightly upregulate the combination of IL-37 and IL-1R8 (p > 0.05 VS control). In the heart tissues of mice in the CVB3 infection group, we found that CVB3 infection significantly up-regulated the binding of IL-37 and IL-1R8. Does CVB3 infection play an anti-inflammatory effect by up-regulating the binding of IL-37 and IL-1R8? Our research team believes that this may be due to the body's protective response (a protective mechanism) in response to CVB3 infection. Not surprisingly, IL-37 treatment further enhances the combination of IL-37 and IL-1R8 on the basis of CVB3 infection, which can be seen as artificially enhancing the human body’s protective mechanism against CVB3. However, it is beyond the scope of this study to examine the cardioprotective function of the complex of IL-37 and IL-1R8. Further experiments in vivo and vitro are needed to explore the consequent signal transduction of IL-37 and IL-1R8.

## Conclusions

In viral myocarditis, in addition to the direct damage to cardiomyocytes caused by virus replication, continuous inflammation is the most important factor leading to the continuous aggravation of viral myocarditis^5,23^. A moderate inflammatory response is indispensable to clear the virus, but an over-activated inflammatory response, such as cytokine storm, can cause more serious heart damage^2^. Therefore, a balance between inflammation and anti-inflammation is very important for the treatment of myocarditis. In the current study, we confirmed that the protective effect of IL-37 on the heart against CVB3 infection may be related to its effect on the NLRP3 inflammasome-GSDMD, NF-κB p65 and IL-37-IL-1R8-IL-18RA complex. Although the relationship between these signal channels and the mechanism by which IL-37 affects fibrous tissue deposition still need further study, the current results have suggested that IL-37 has an important potential as a treatment for viral myocarditis.

## Supplementary Information

Supplementary Information 1.

Supplementary Information 2.

Supplementary Information 3.

Supplementary Information 4.

Supplementary Information 5.

Supplementary Information 6.

Supplementary Information 7.

## Data Availability

The datasets used or analysed during the current study are available from the corresponding author on reasonable request.
