# Supplementary figures and images for: IL-37 alleviates Coxsackievirus B3-induced viral myocarditis via inhibiting NLRP3 inflammasome-mediated pyroptosis
Source: Sci Rep. 2022 Nov 22;12:20077. doi: 10.1038/s41598-022-22617-y (PMC9684492; doi:10.1038/s41598-022-22617-y)

2020-8-18


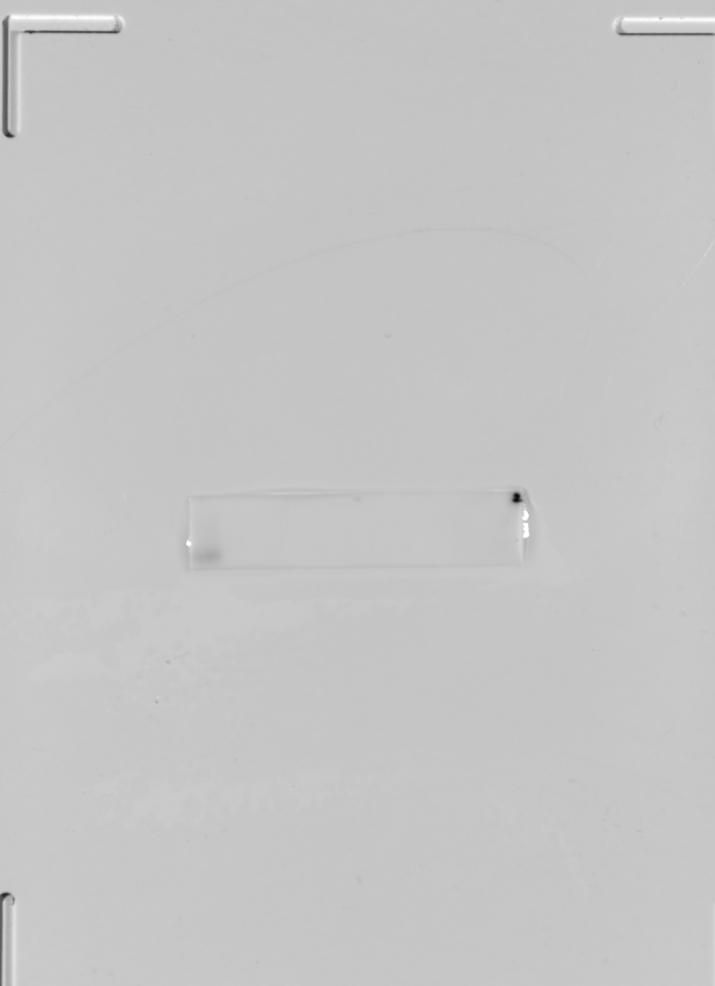

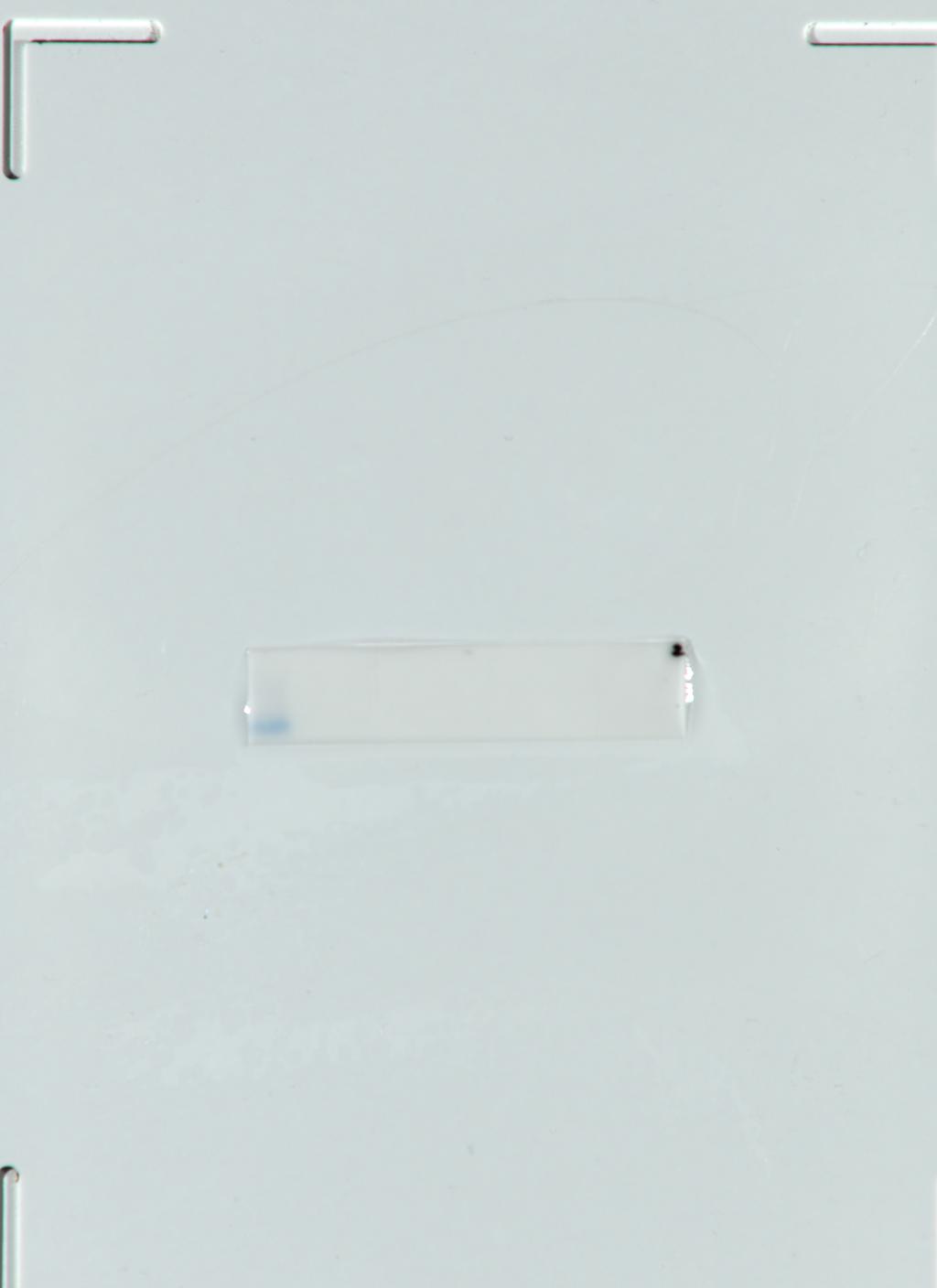

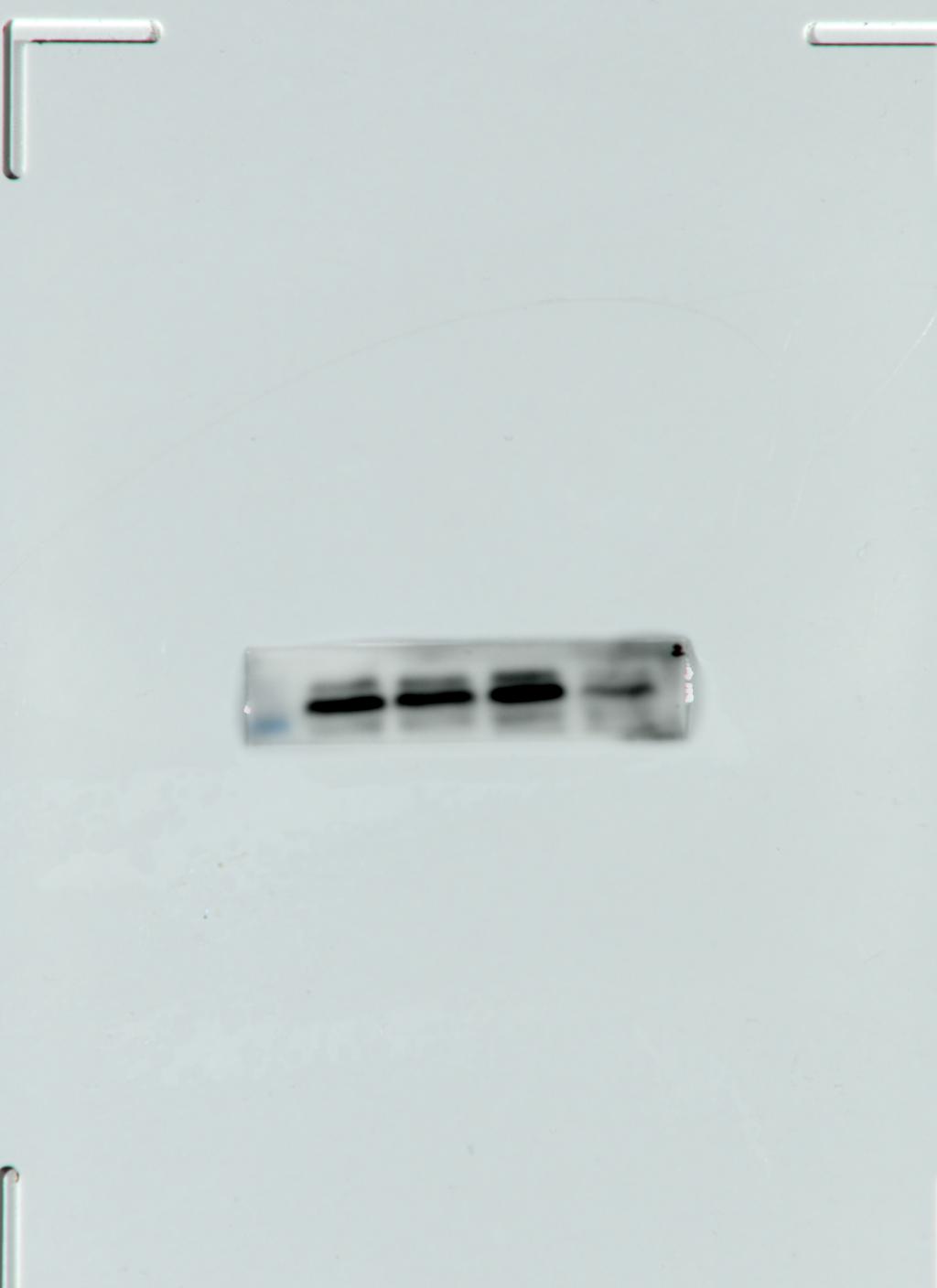


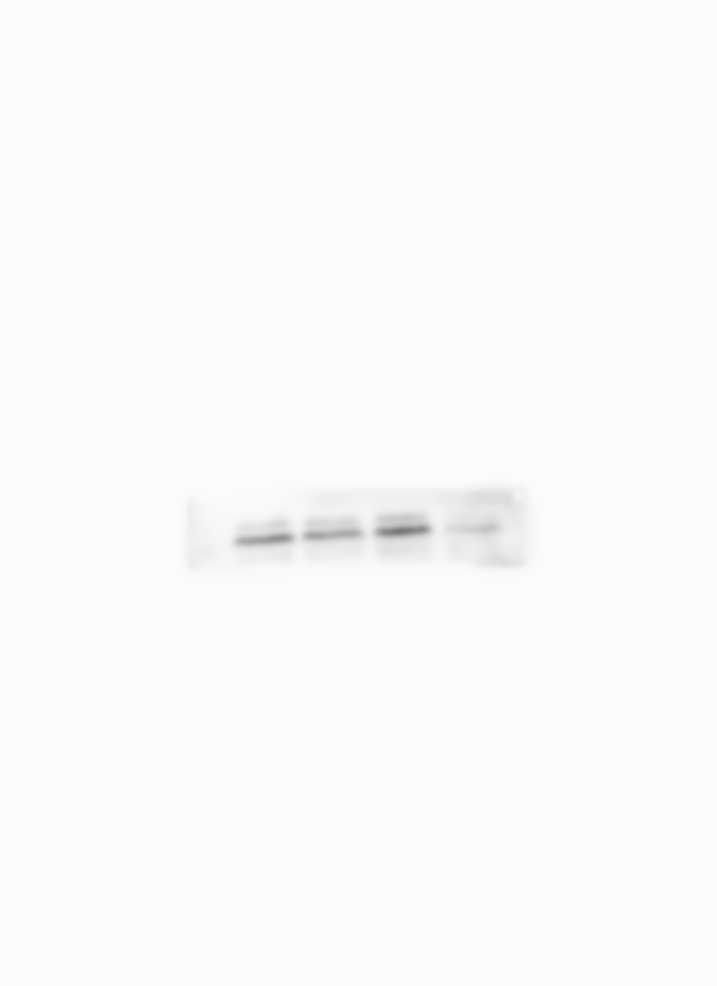


2020-8-21


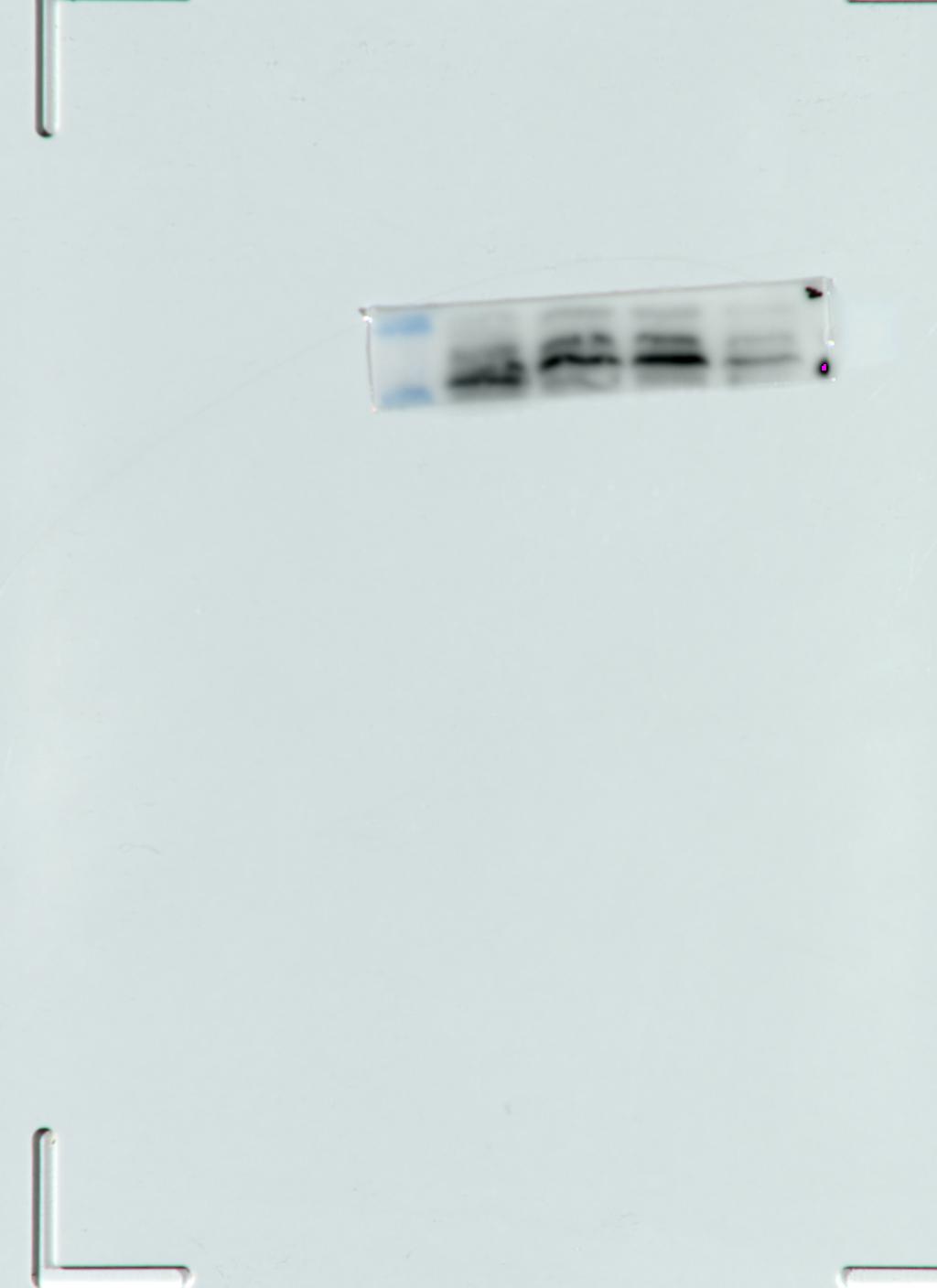

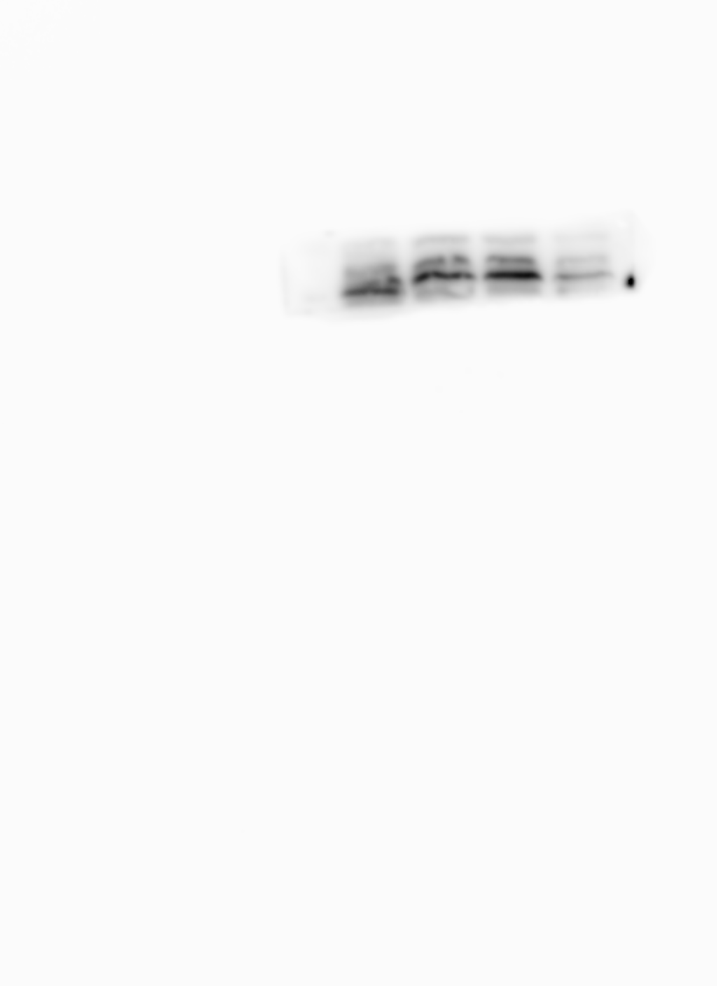


2020-8-27


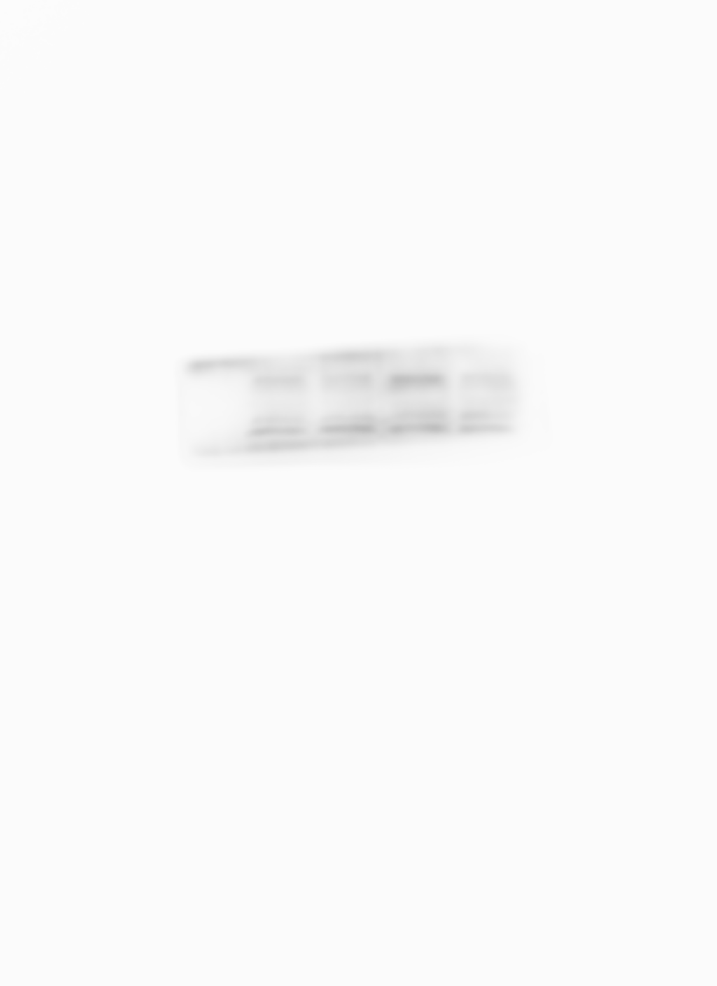

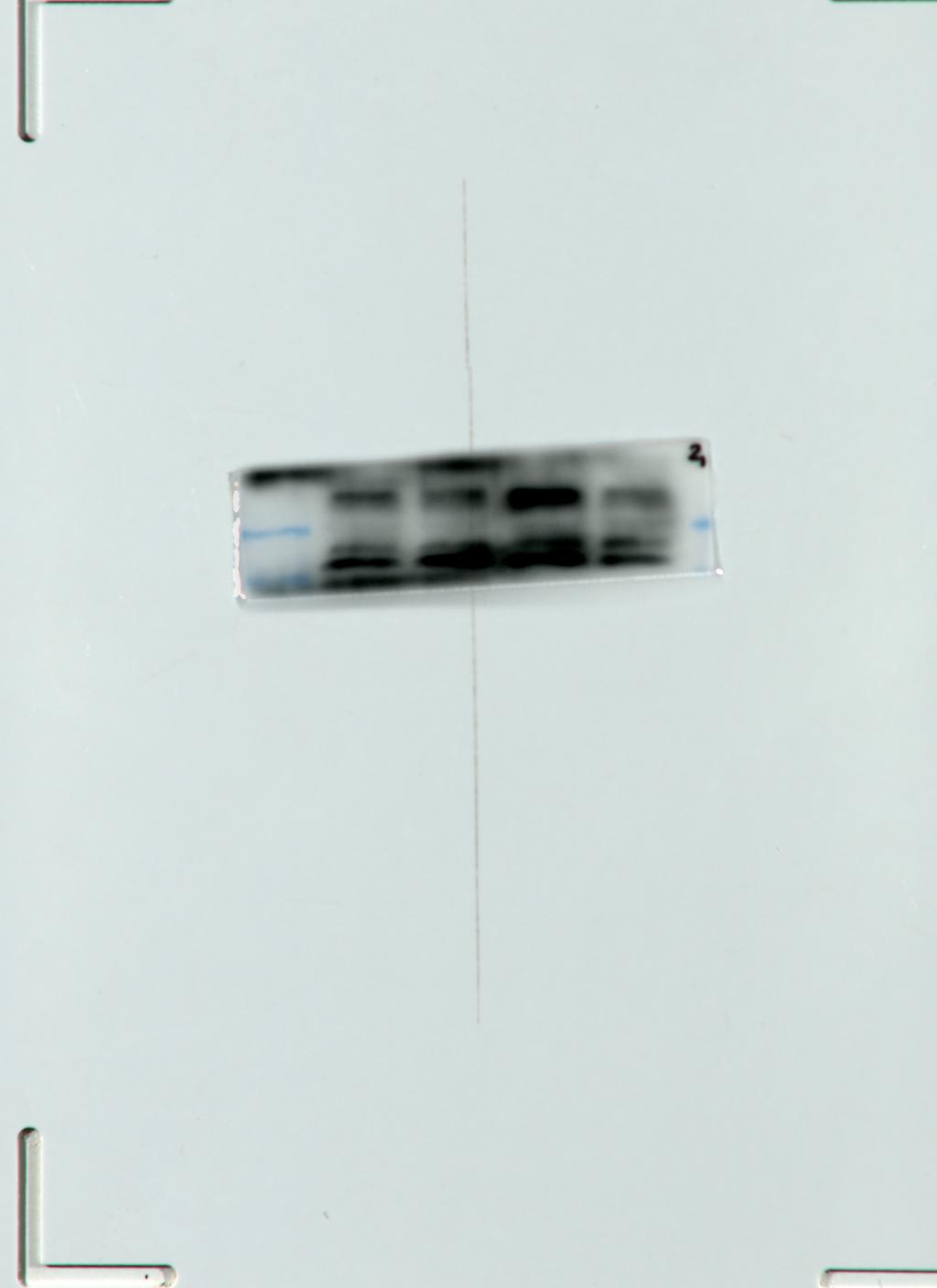

Supplement: Supplementary file 1 — Supplementary Information 1. [file 41598_2022_22617_MOESM1_ESM.docx]

2020-8-18


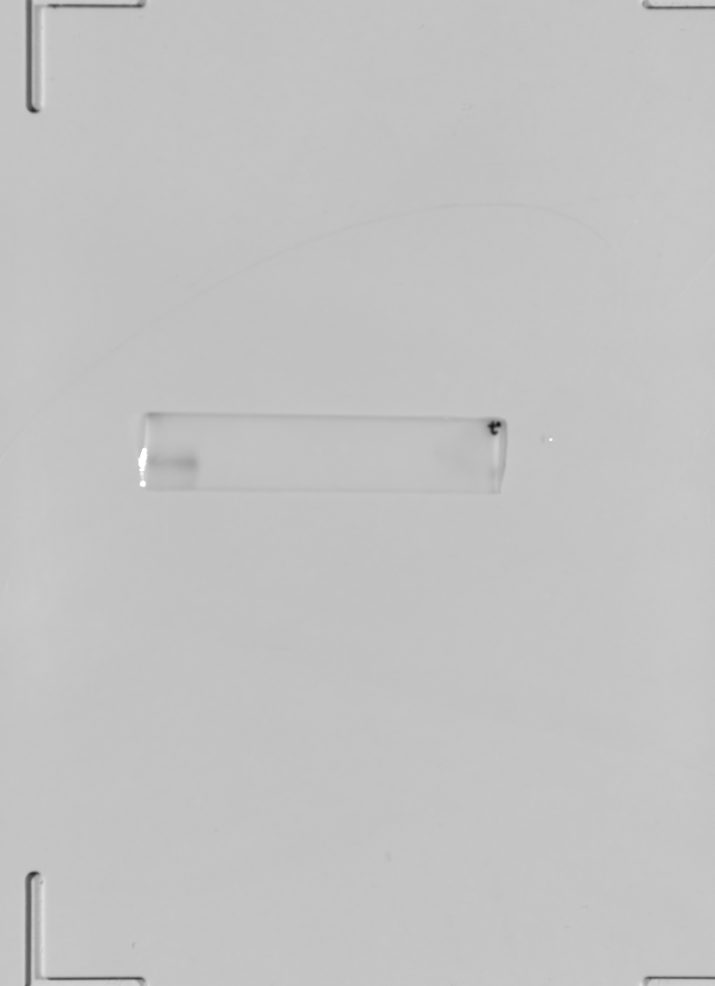

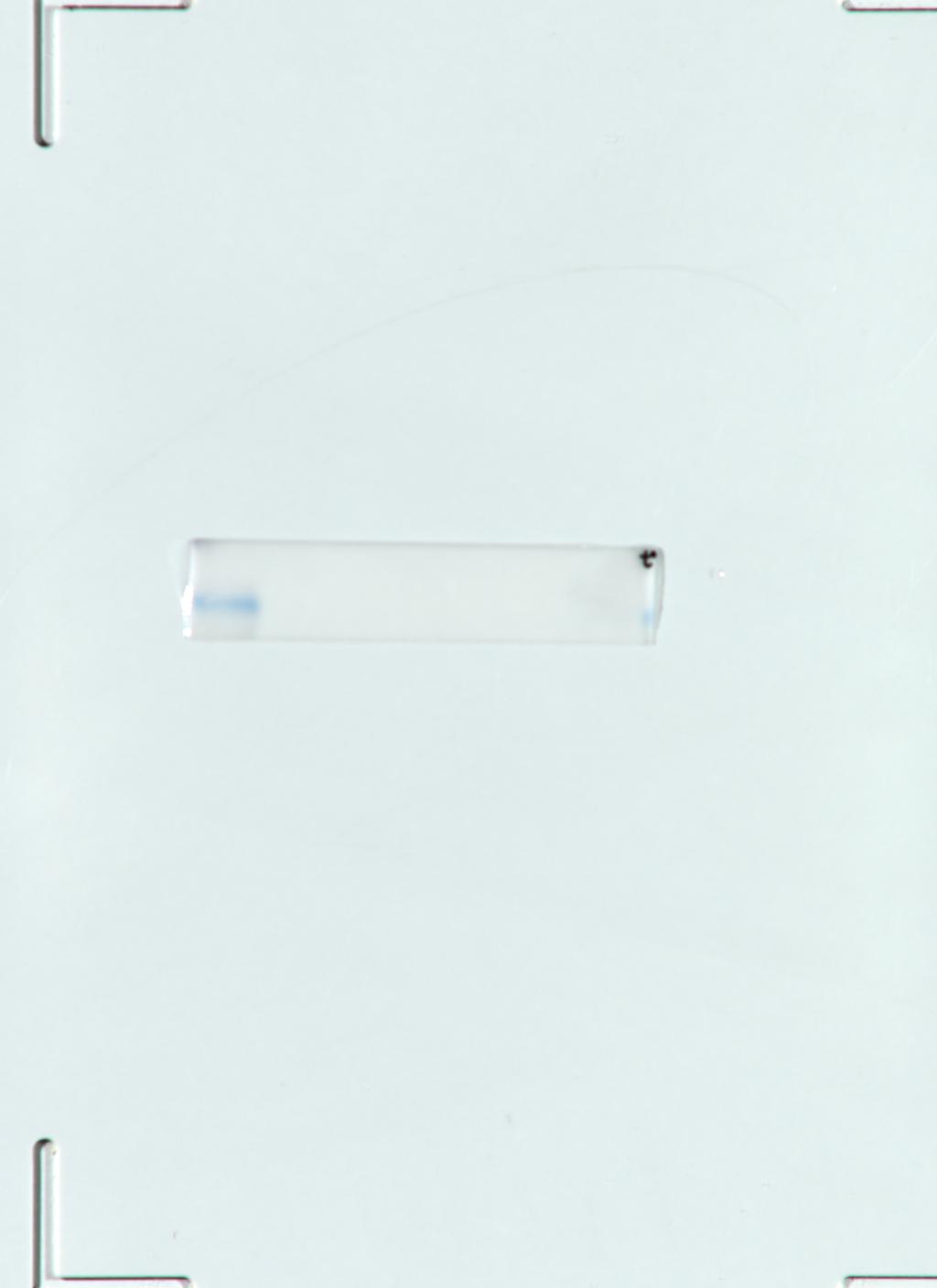

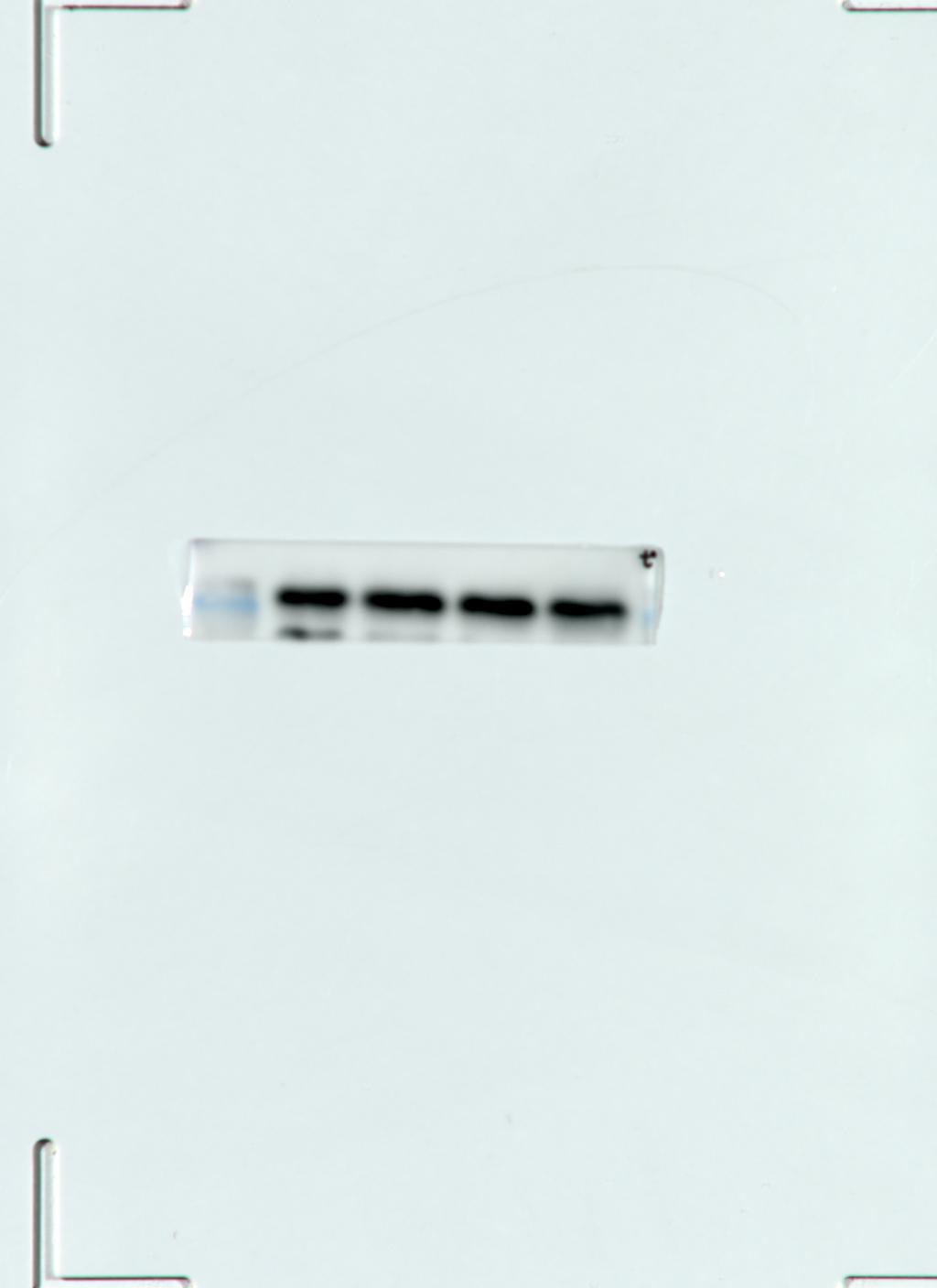

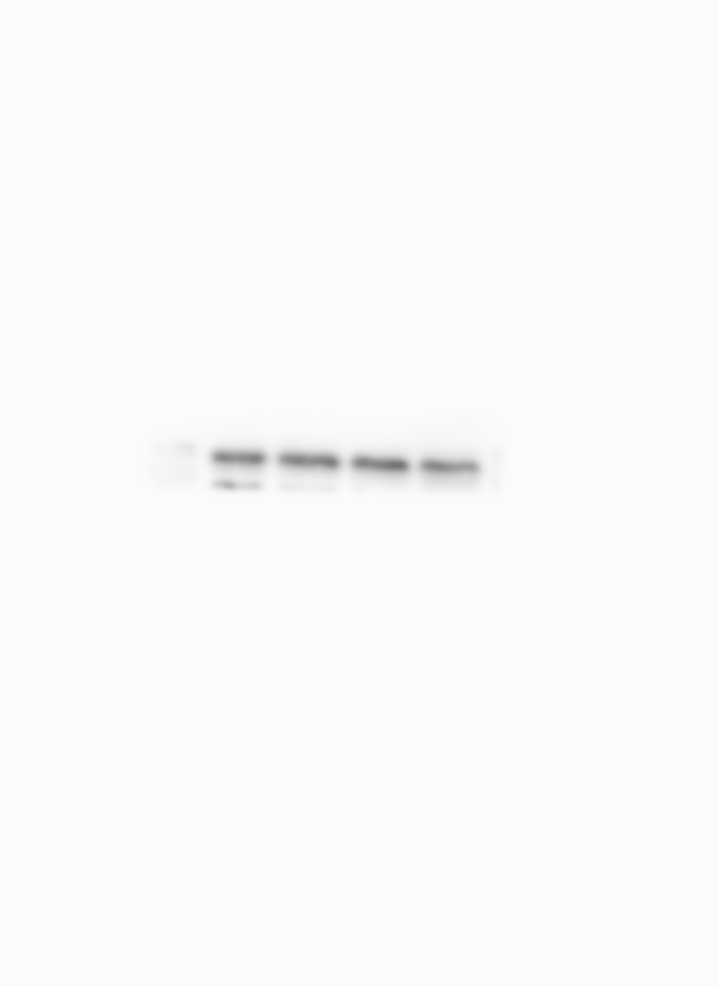


2020-8-21


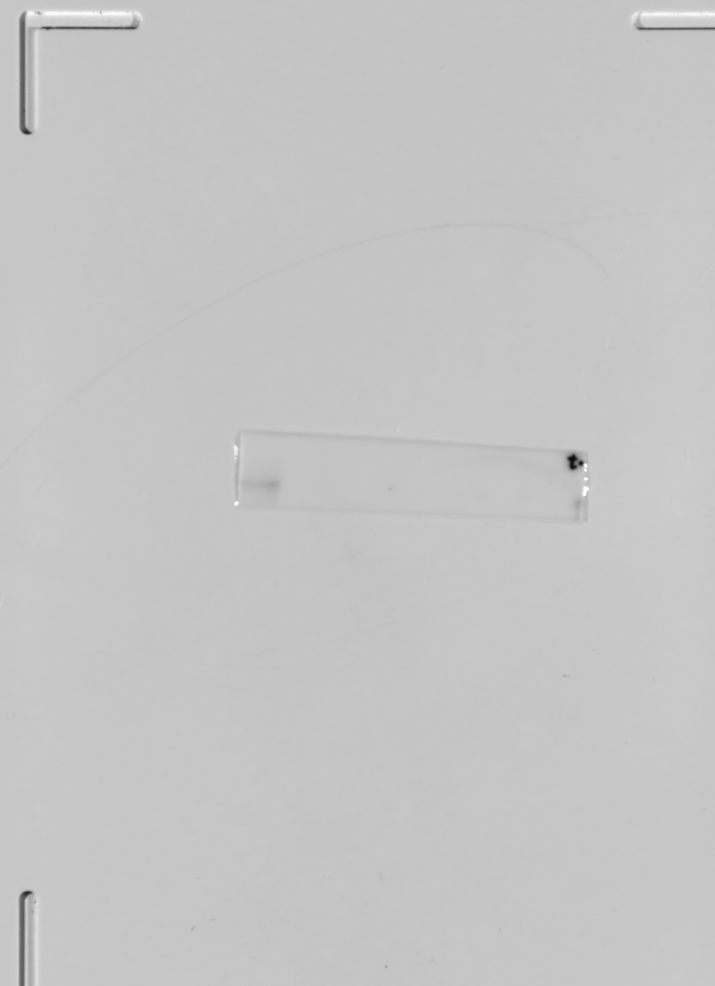

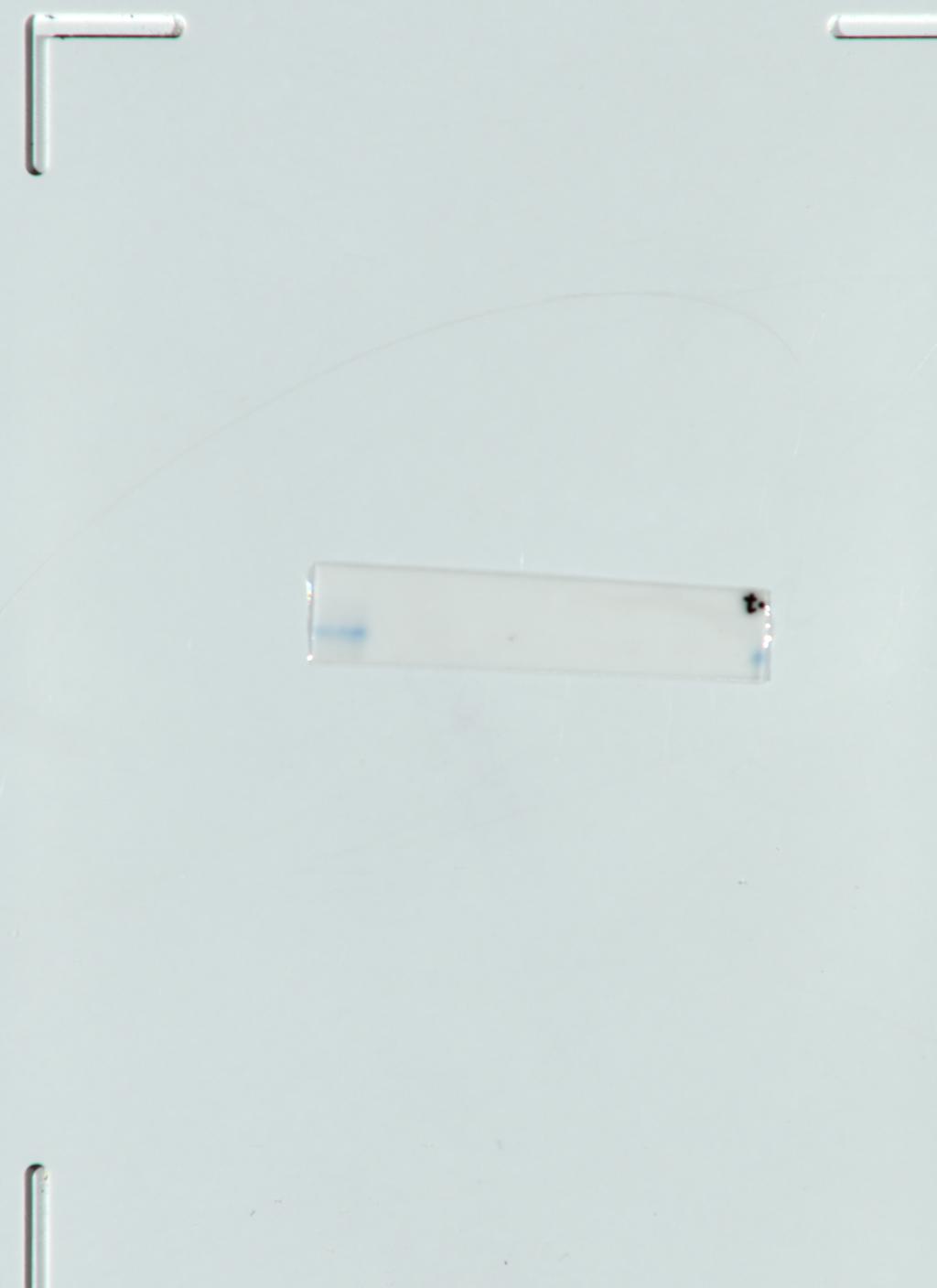

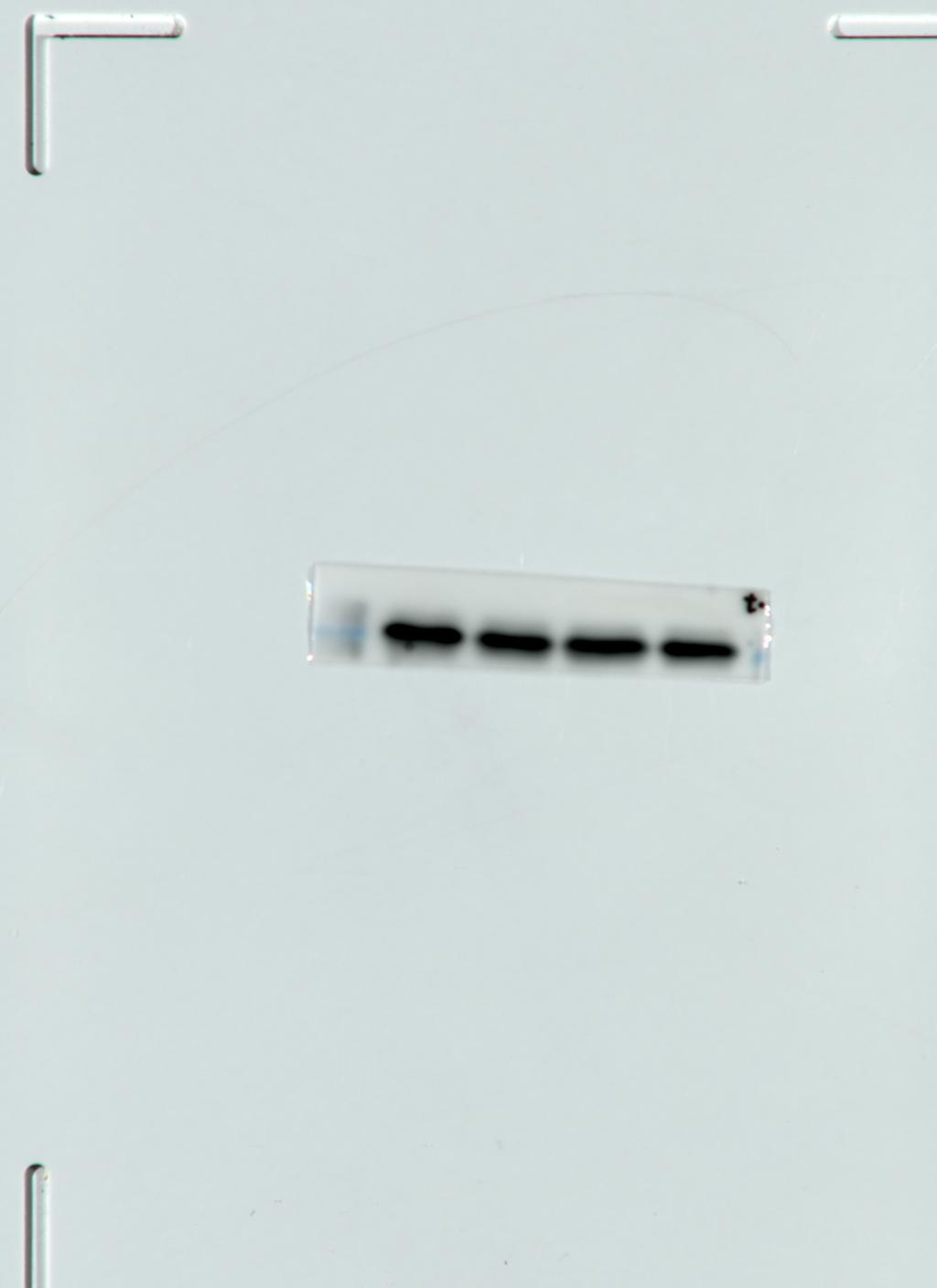

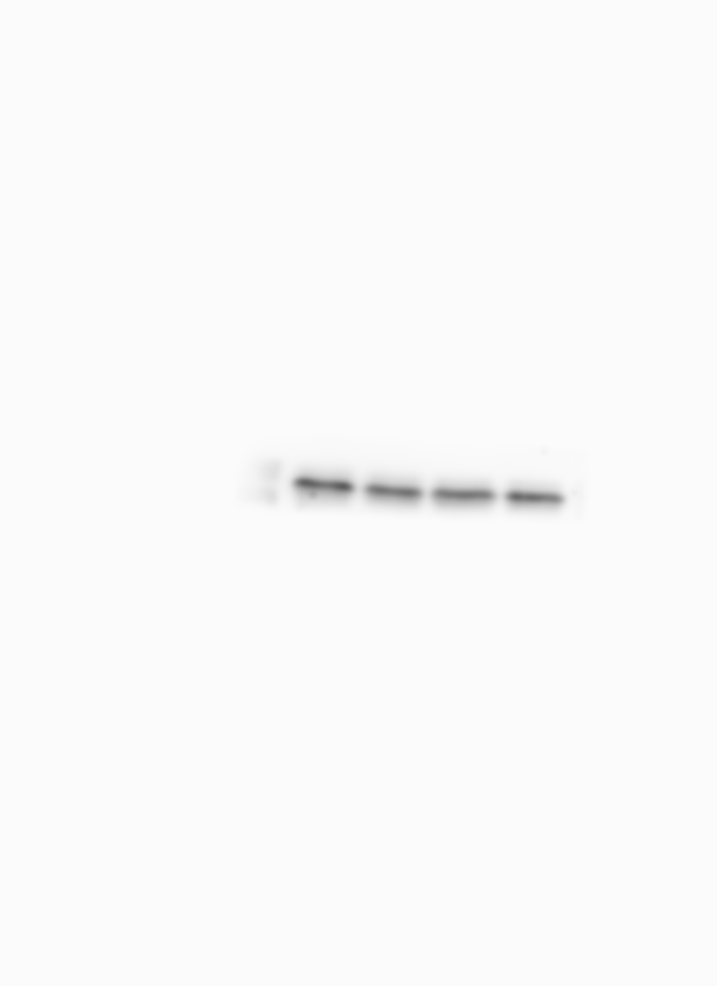


2020-8-27


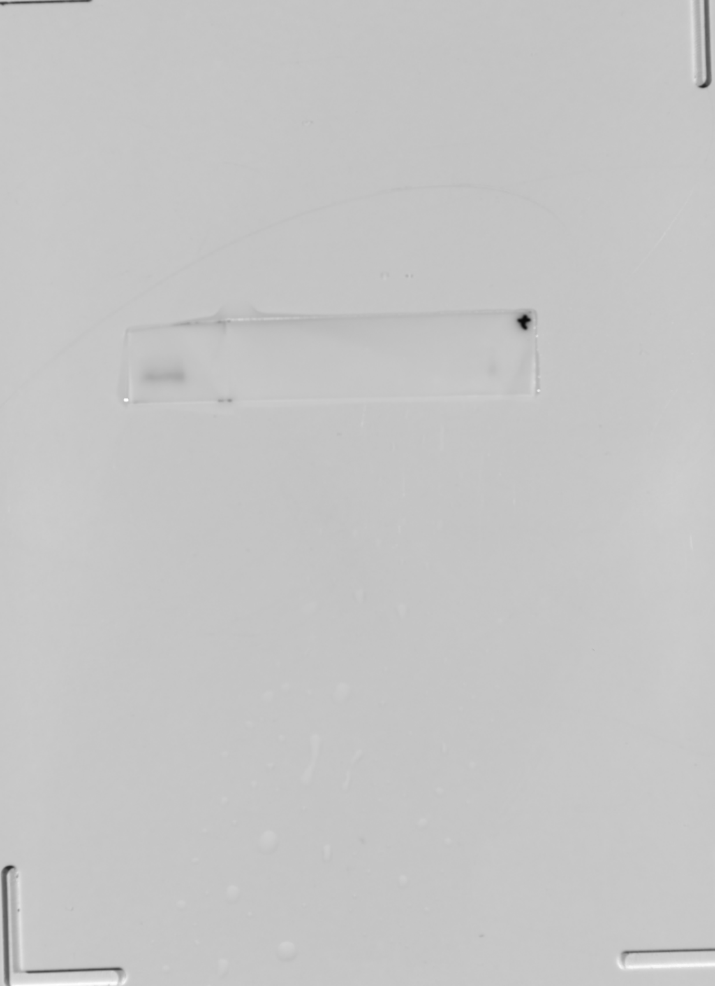

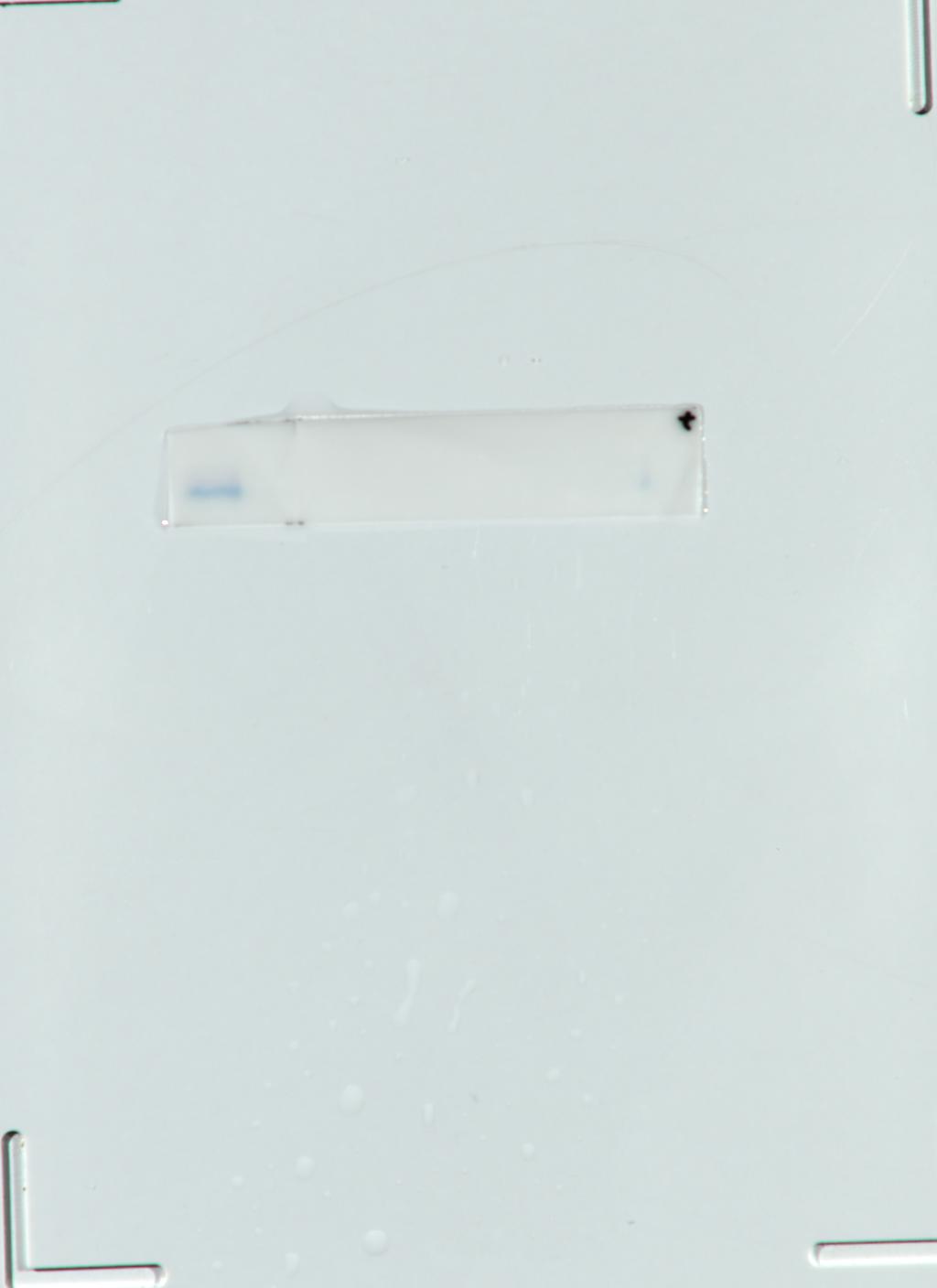

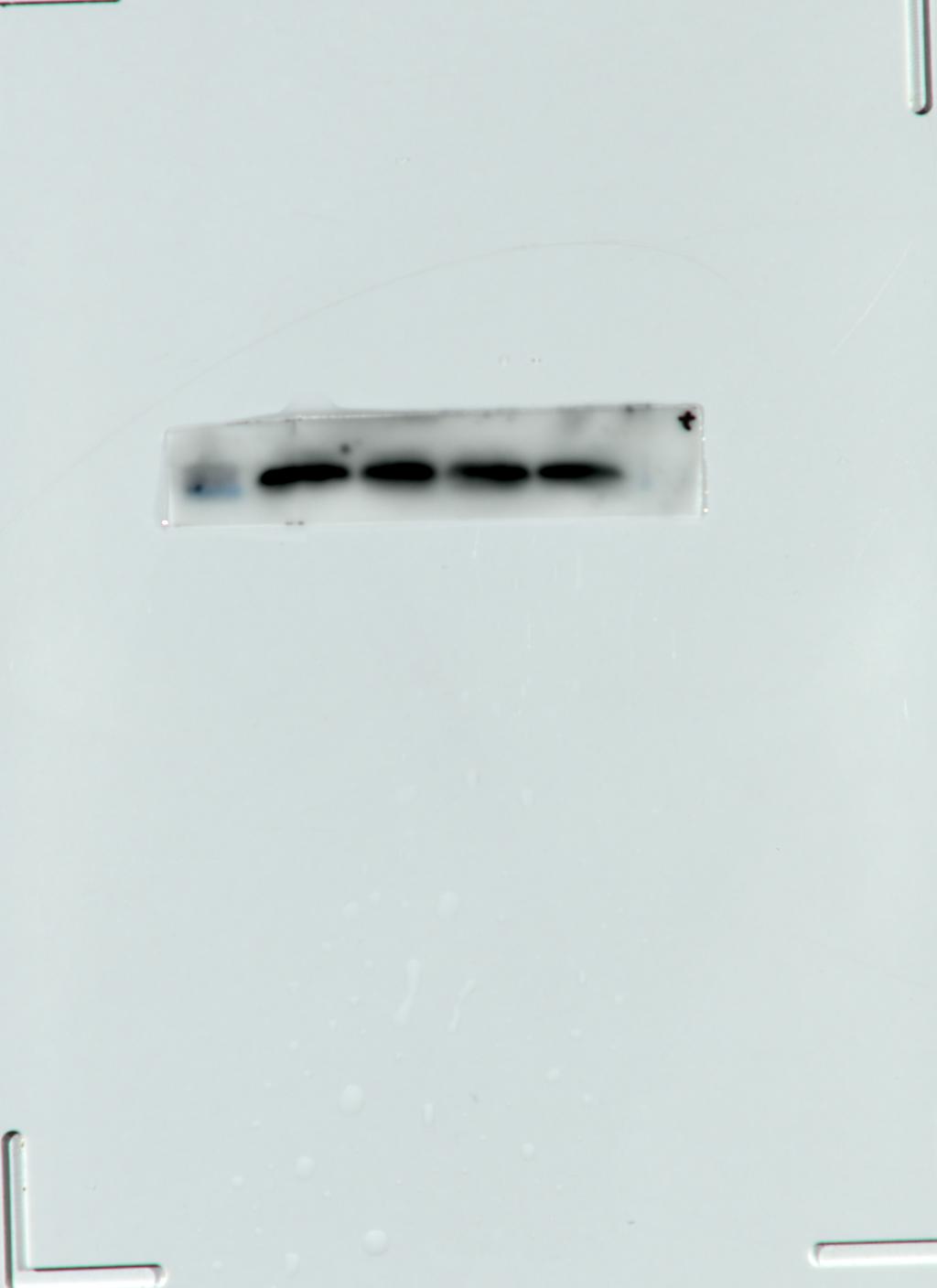

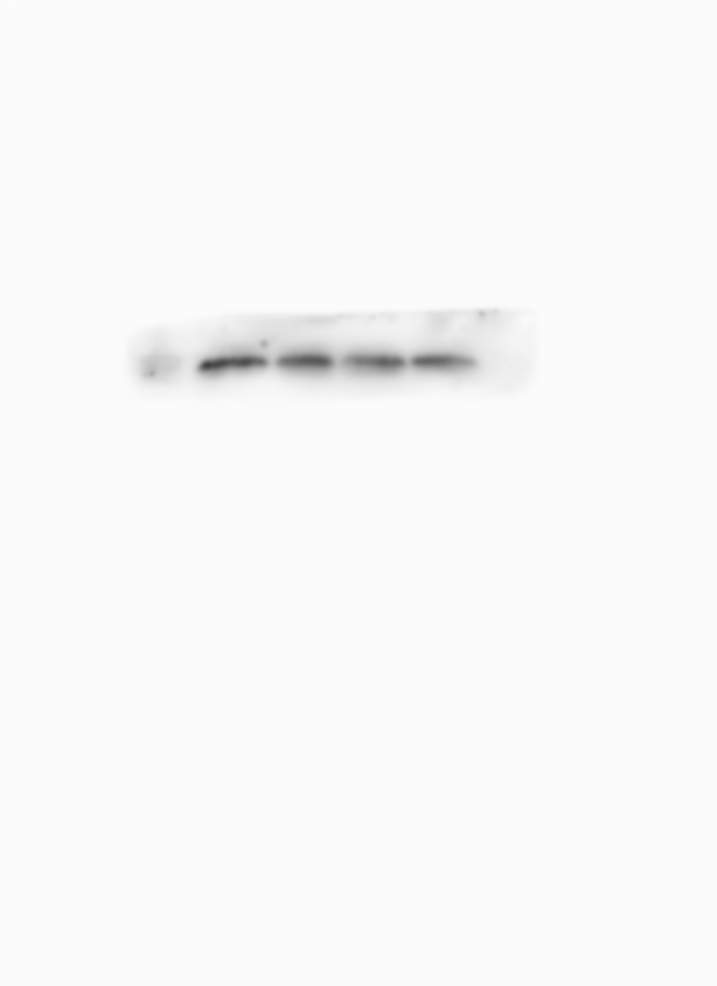

Supplement: Supplementary file 2 — Supplementary Information 2. [file 41598_2022_22617_MOESM2_ESM.docx]

2020-8-18


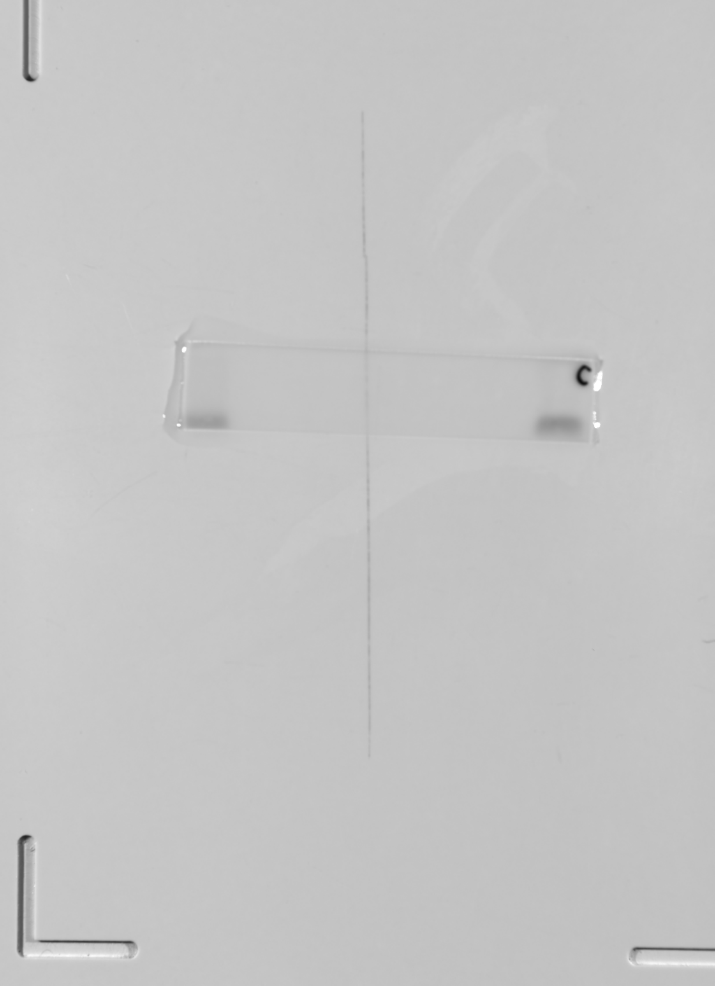

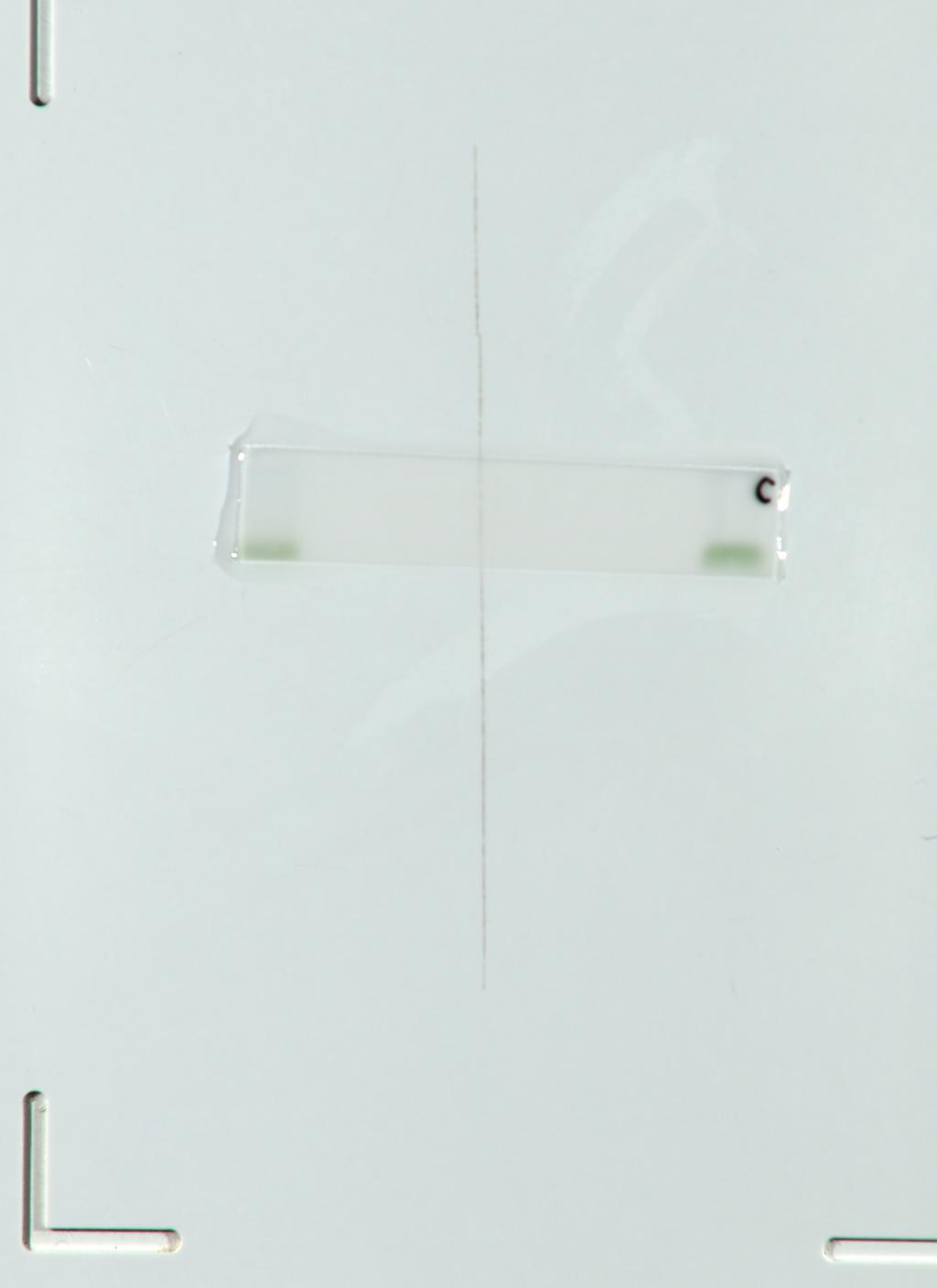

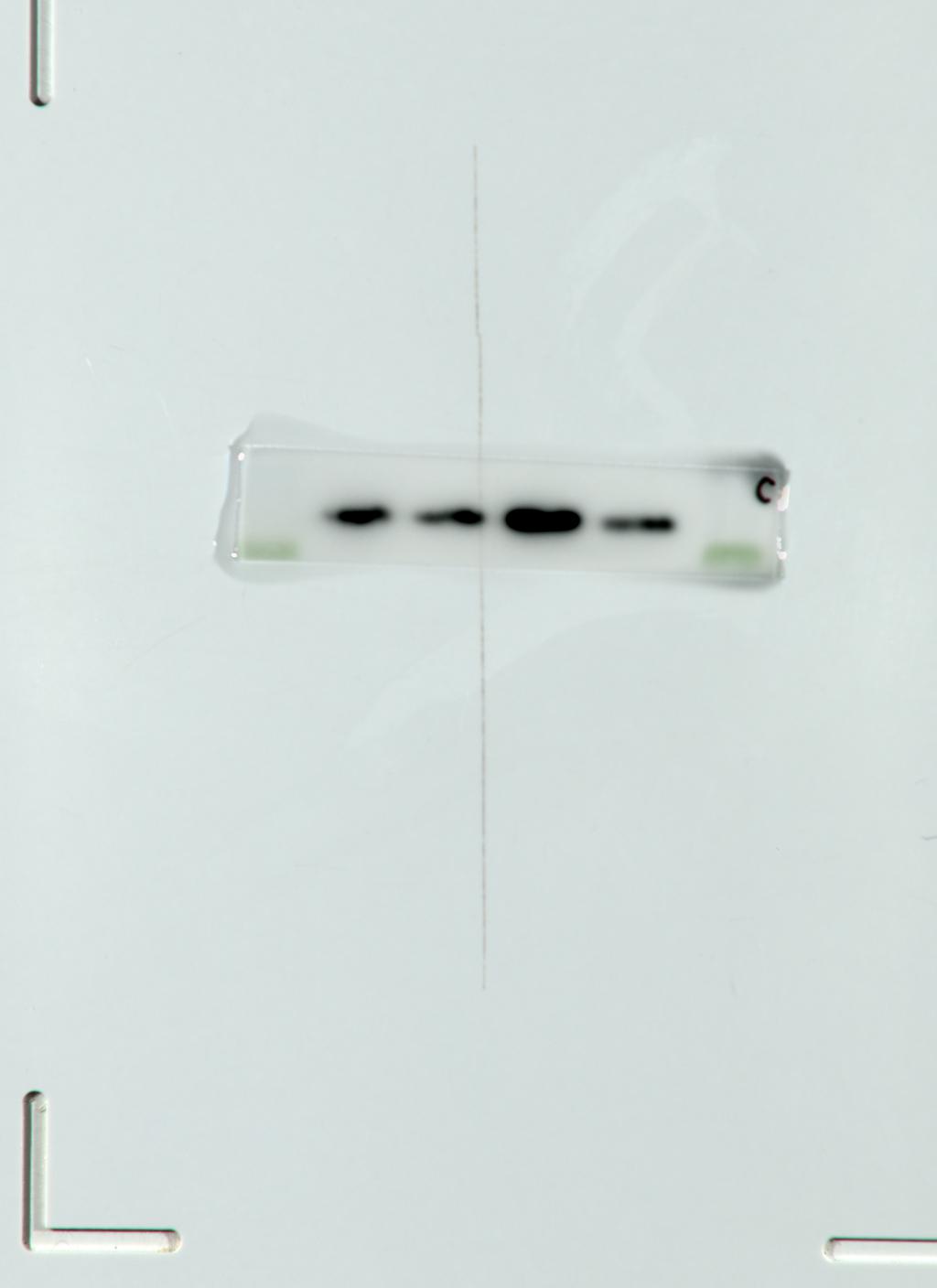

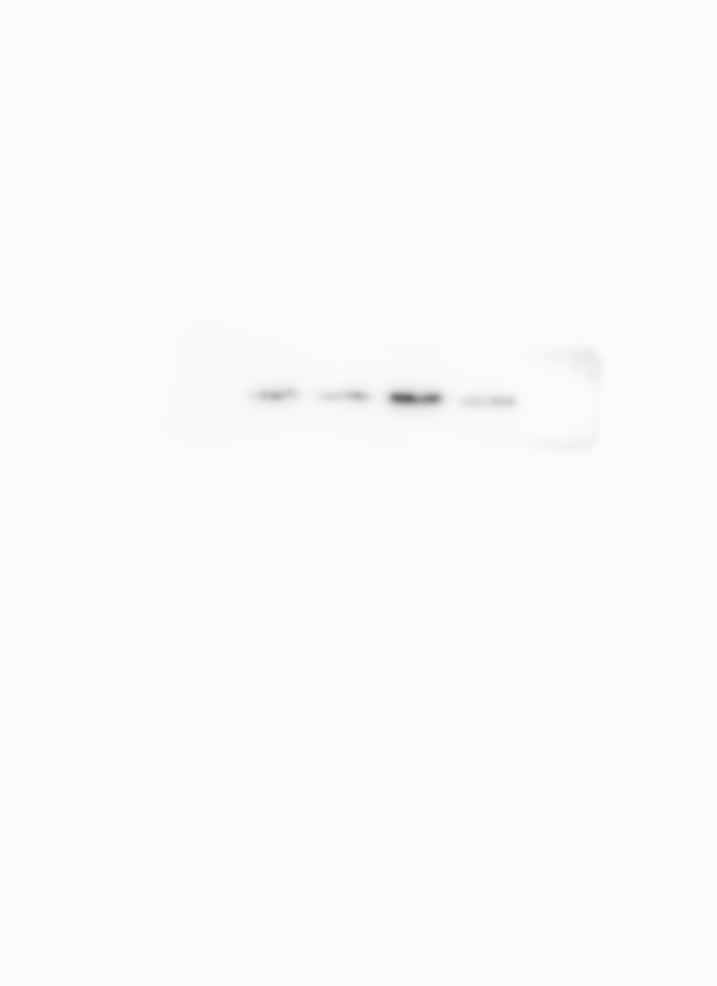

Supplement: Supplementary file 3 — Supplementary Information 3. [file 41598_2022_22617_MOESM3_ESM.docx]

2020-8-18


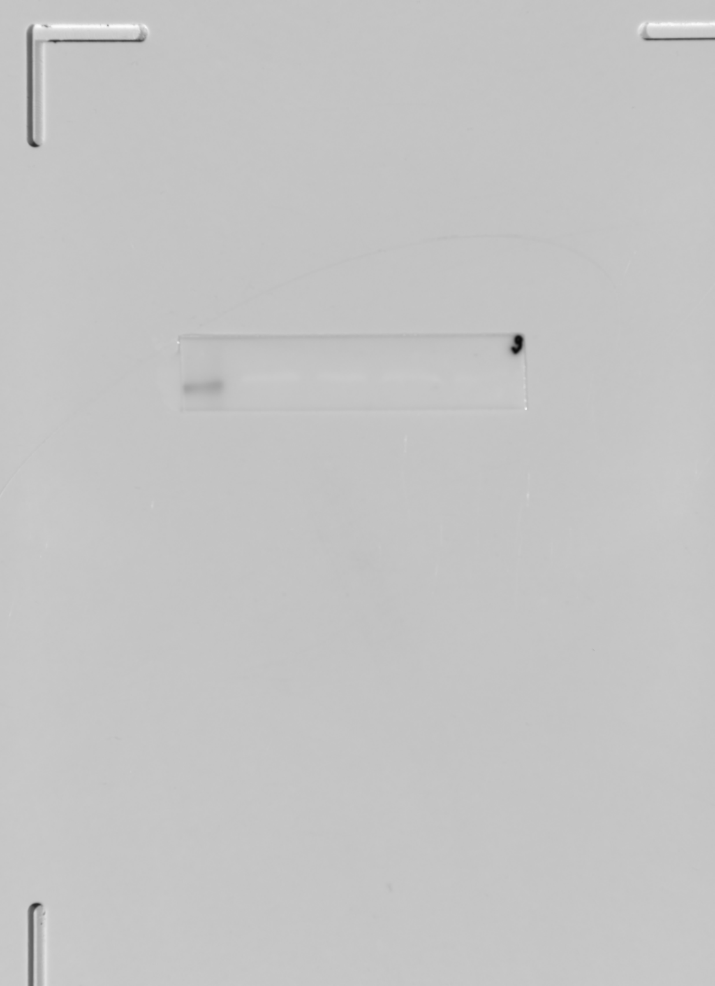

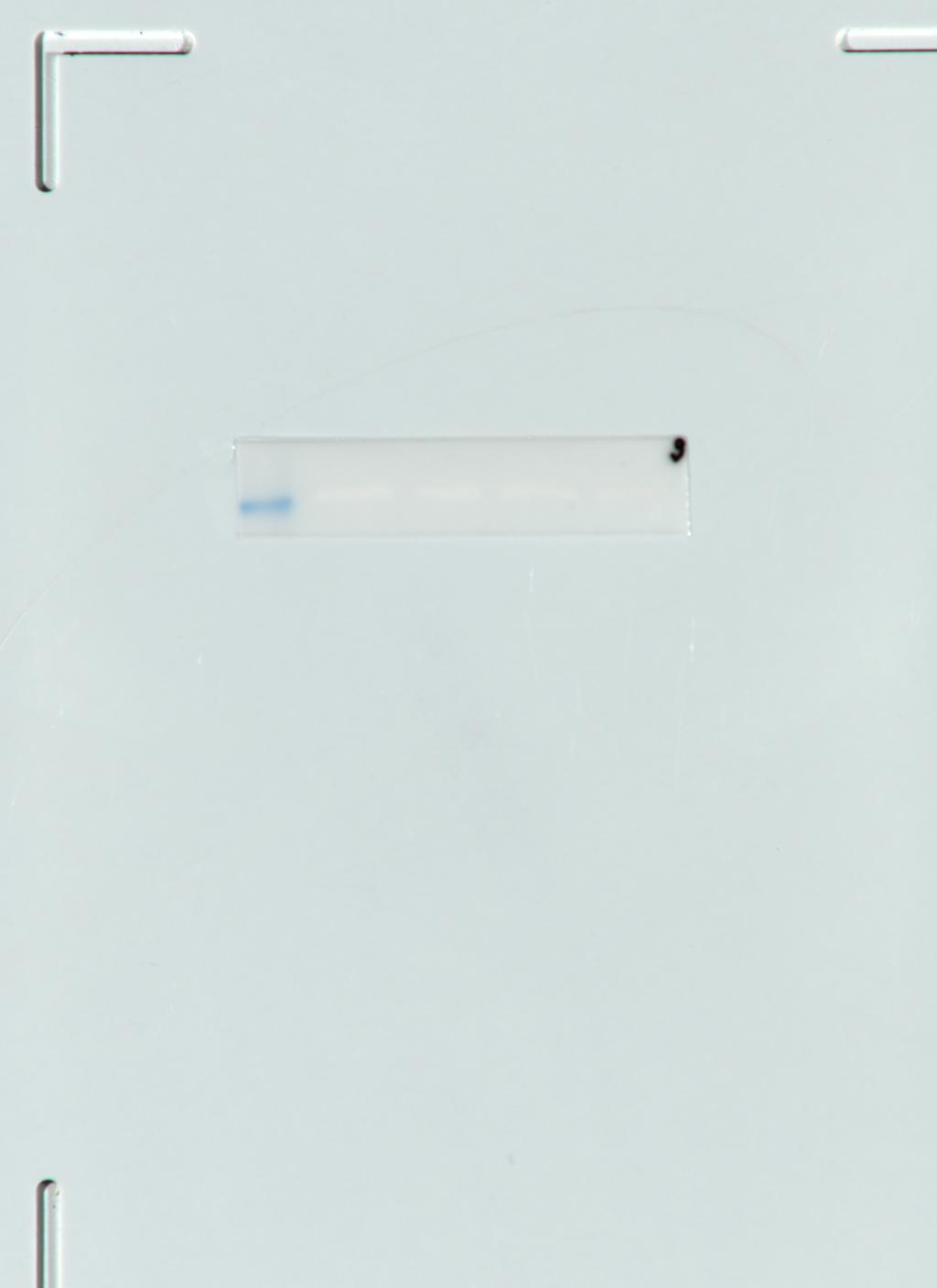

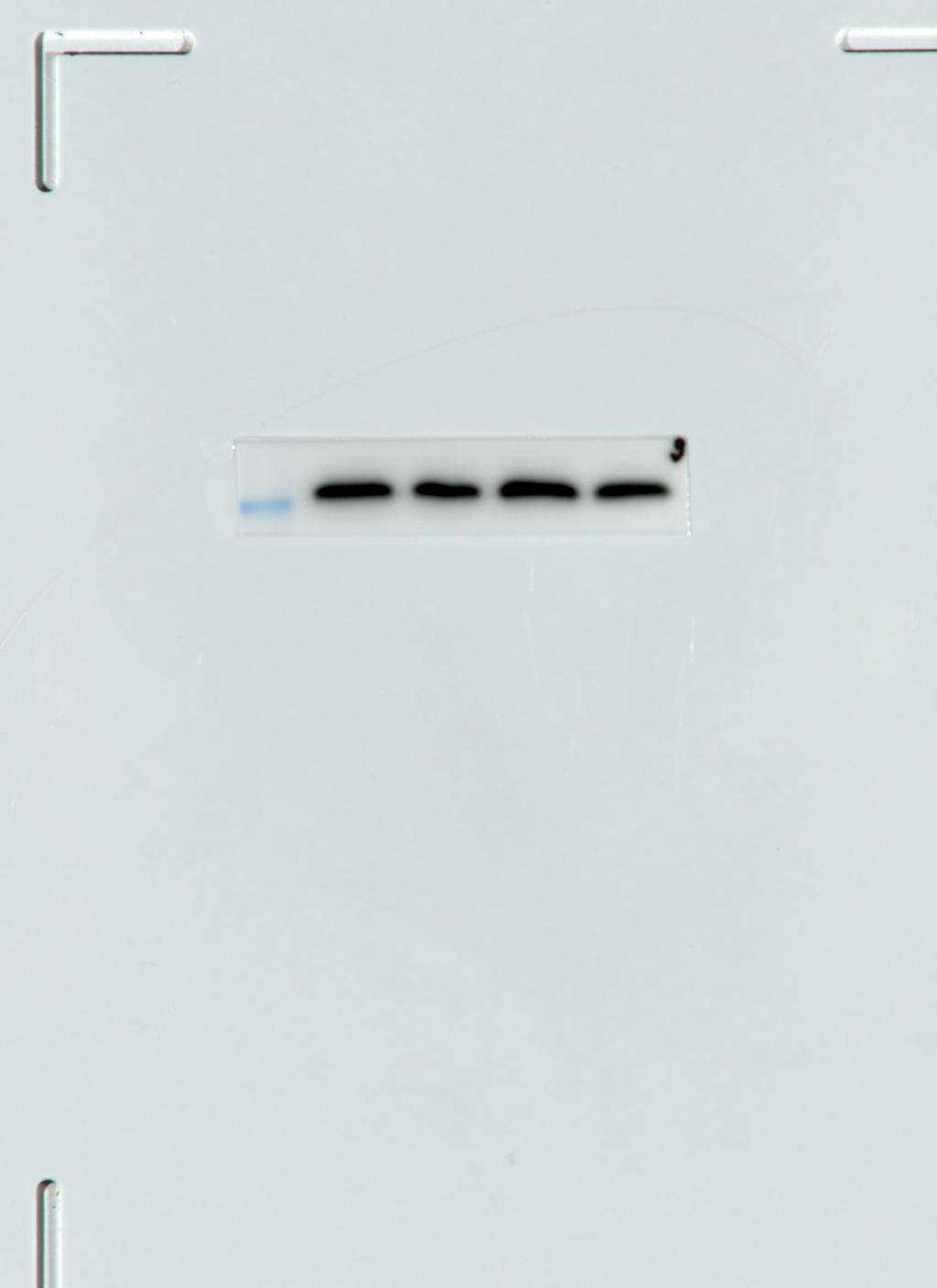

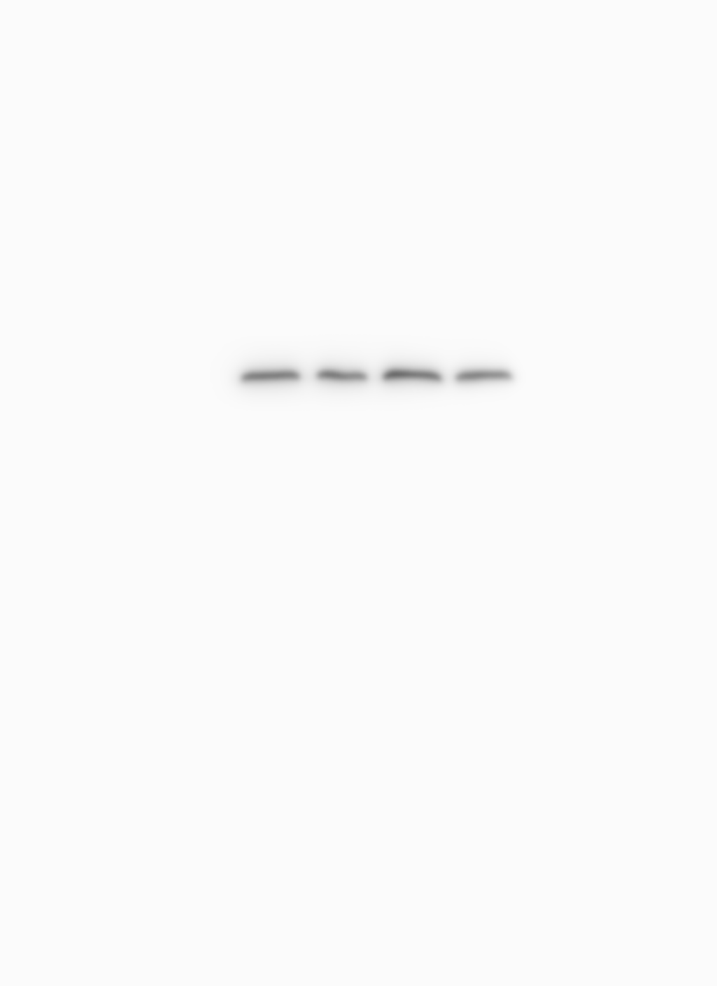


2020-8-21


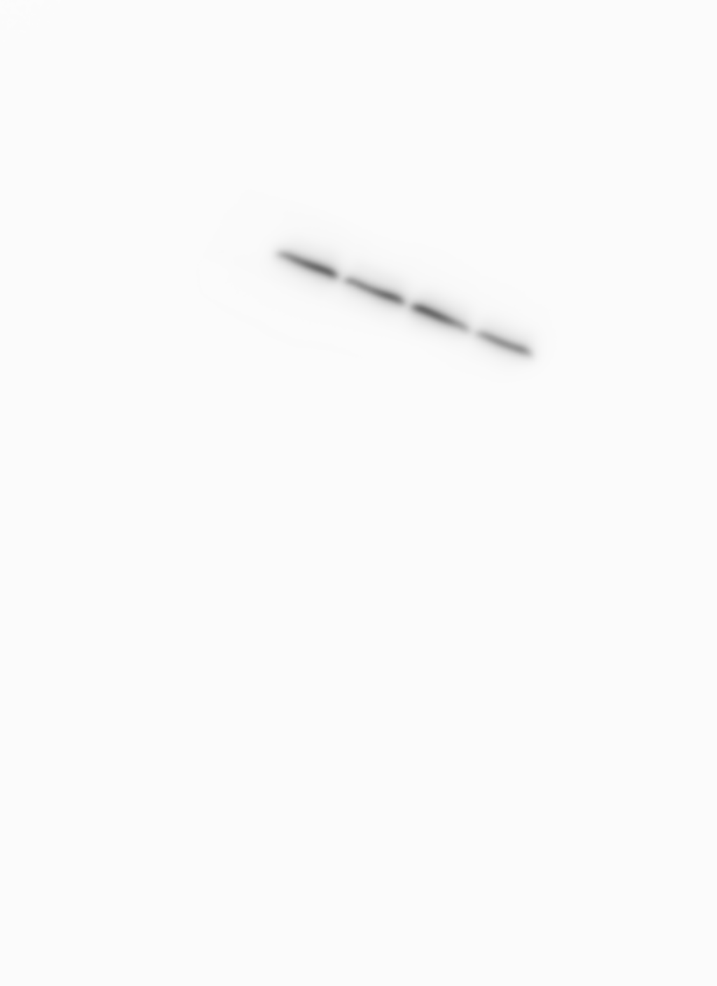

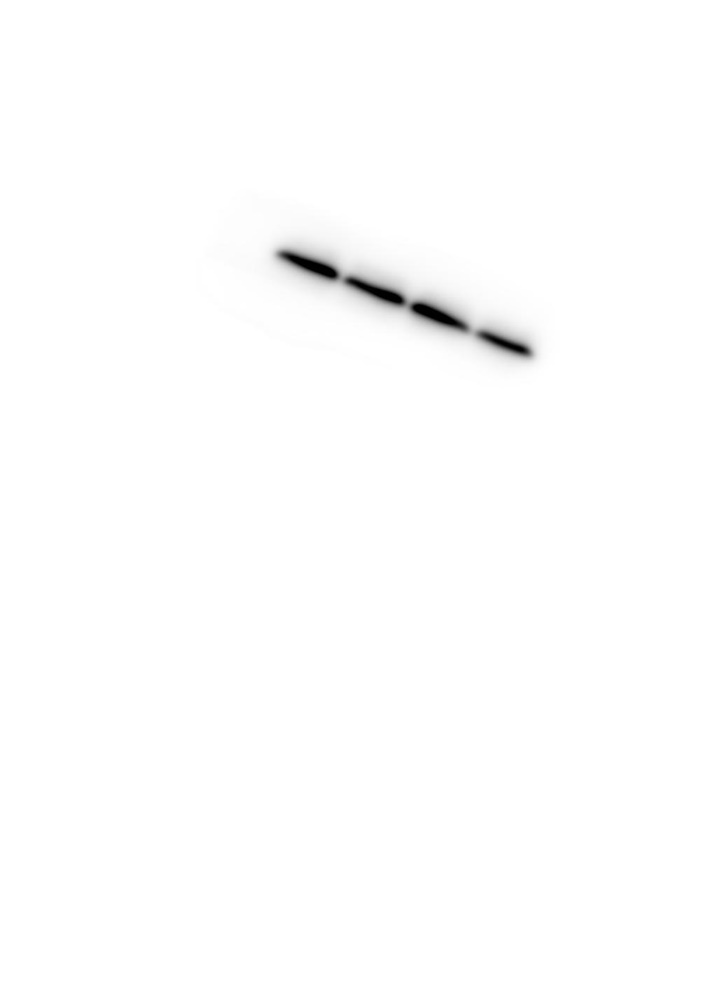


2020-8-27


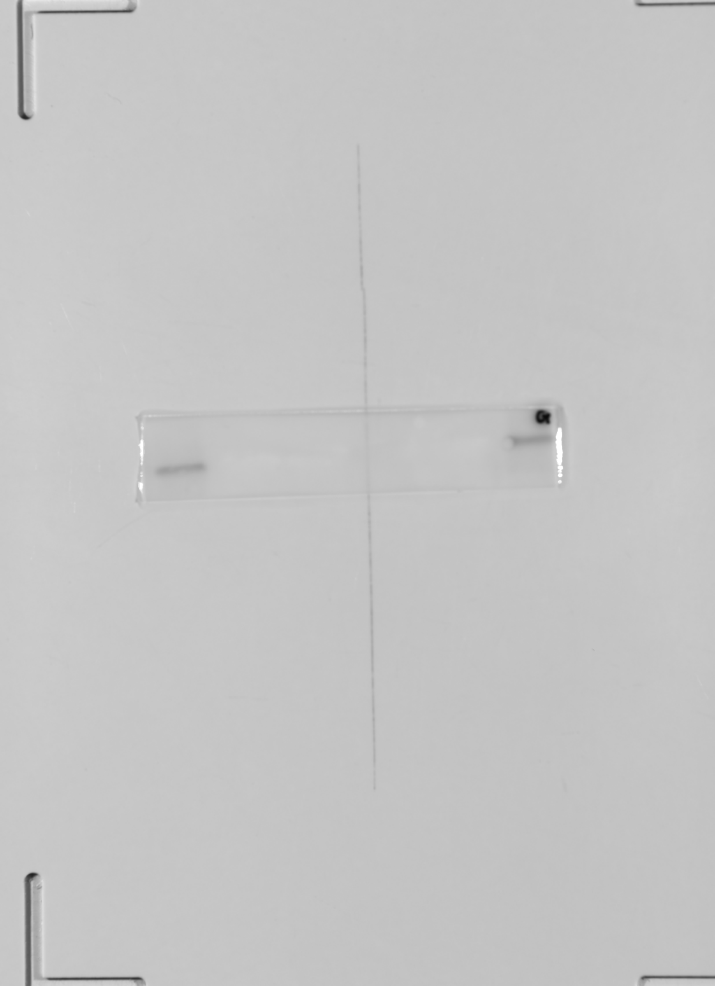

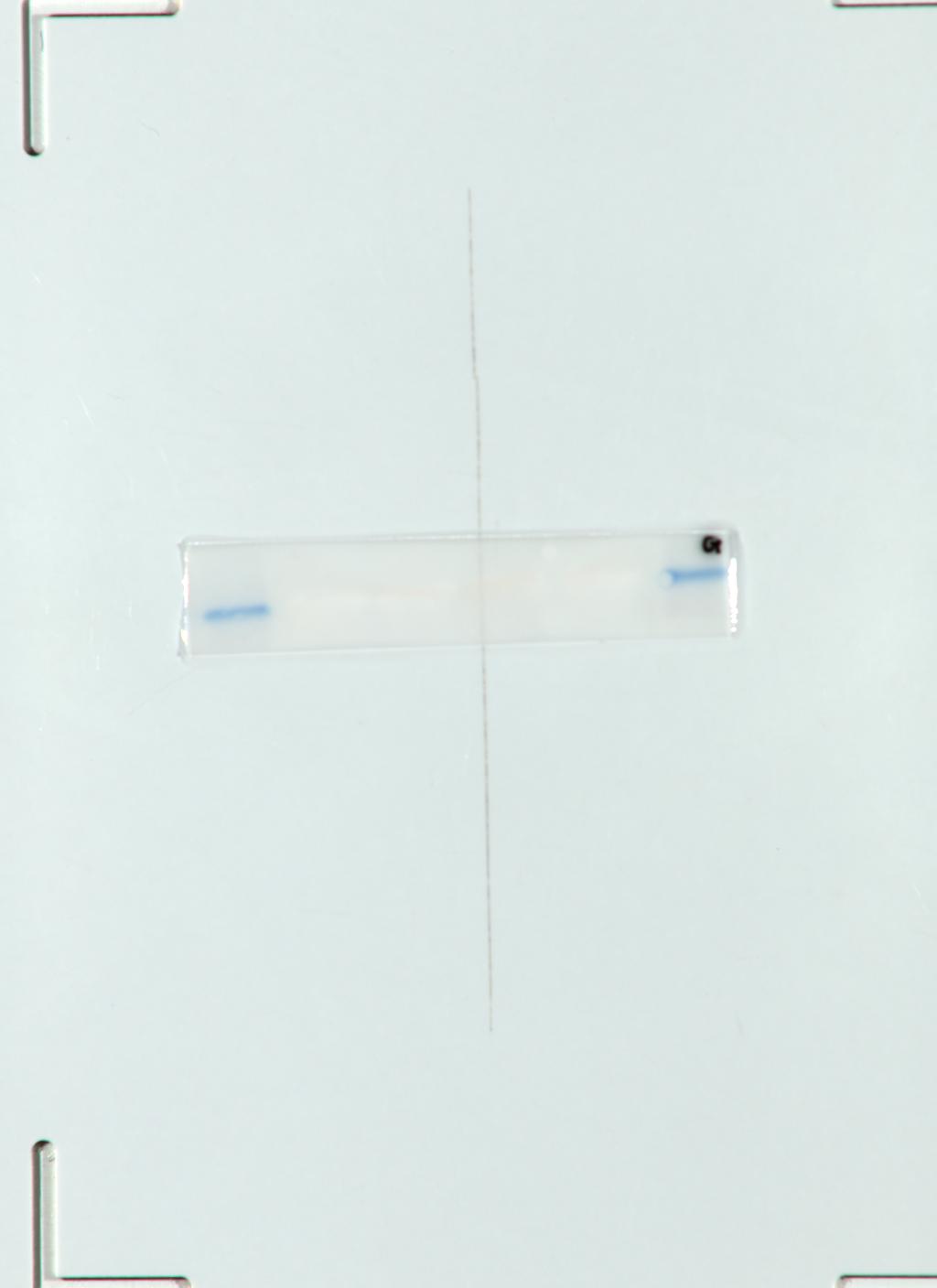

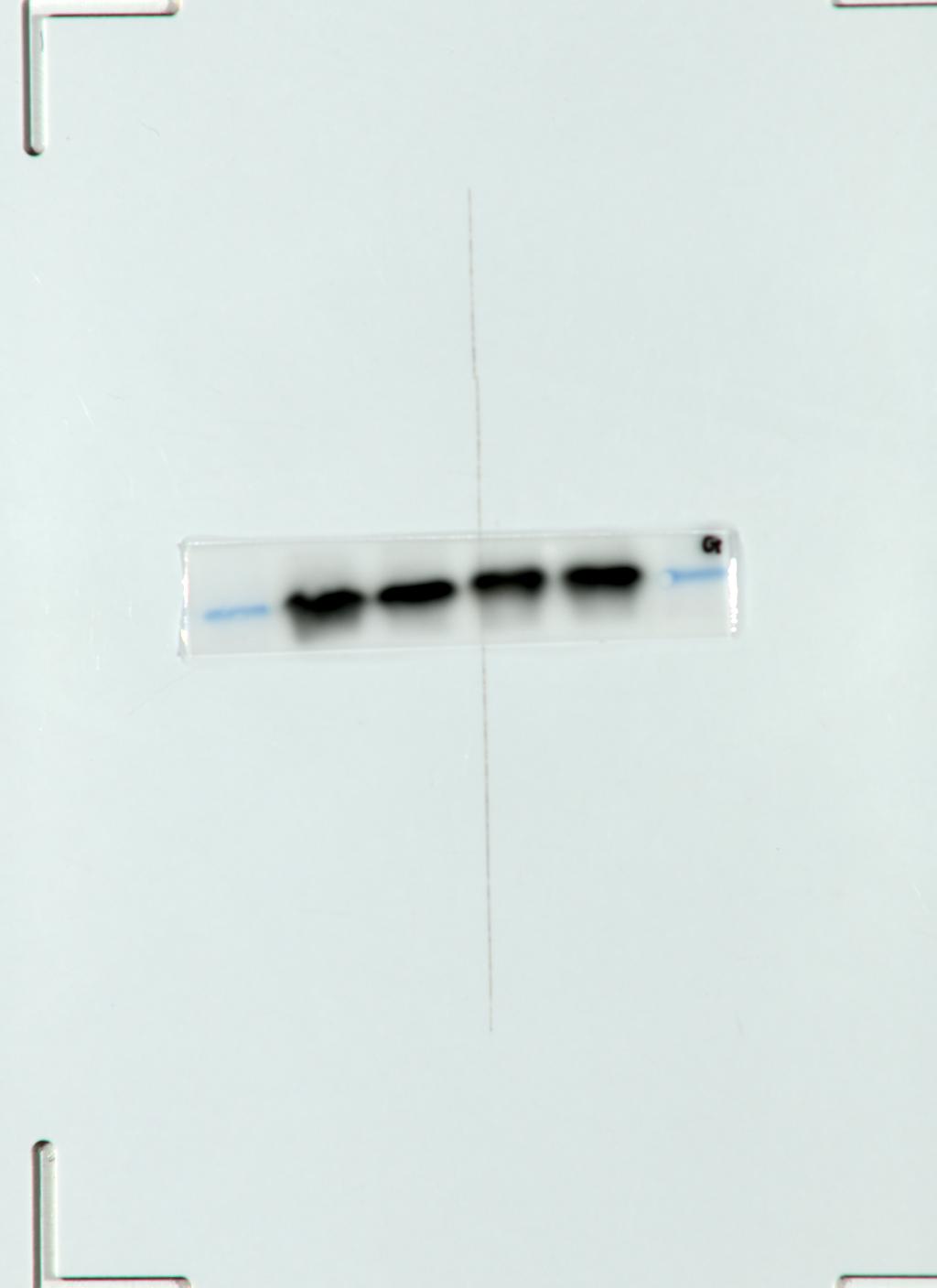

Supplement: Supplementary file 4 — Supplementary Information 4. [file 41598_2022_22617_MOESM4_ESM.docx]

2020-8-18


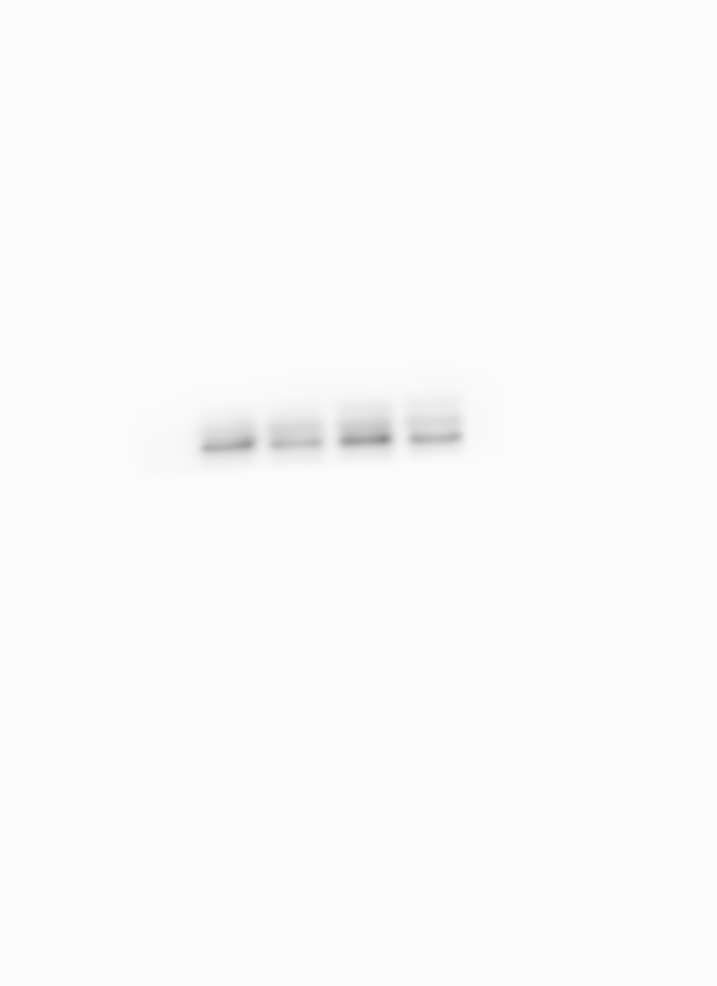

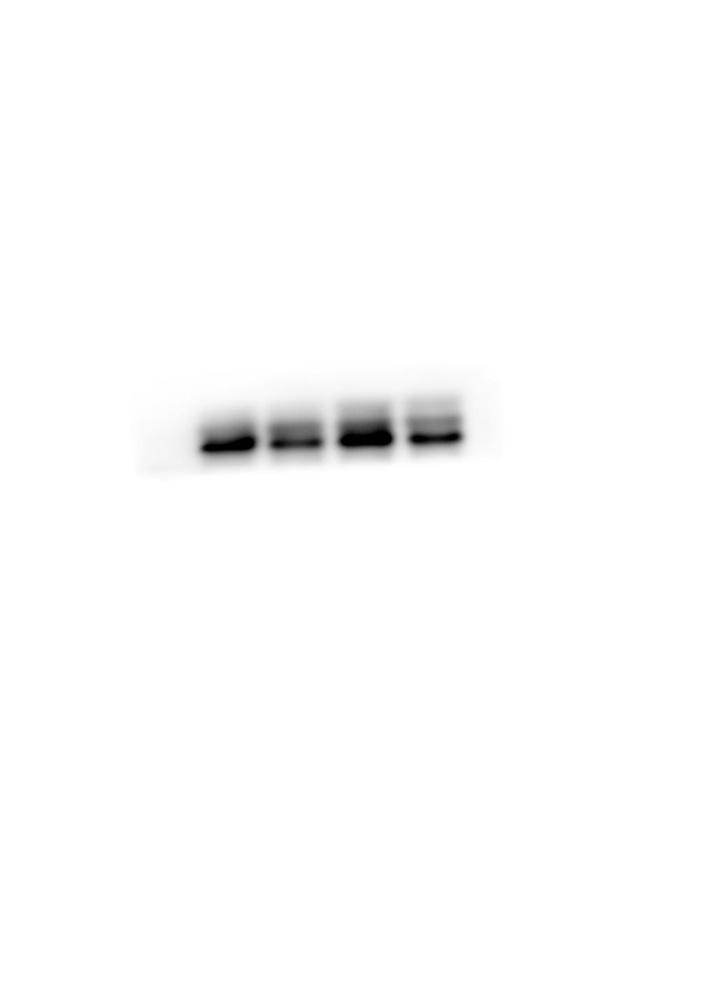


2020-8-21


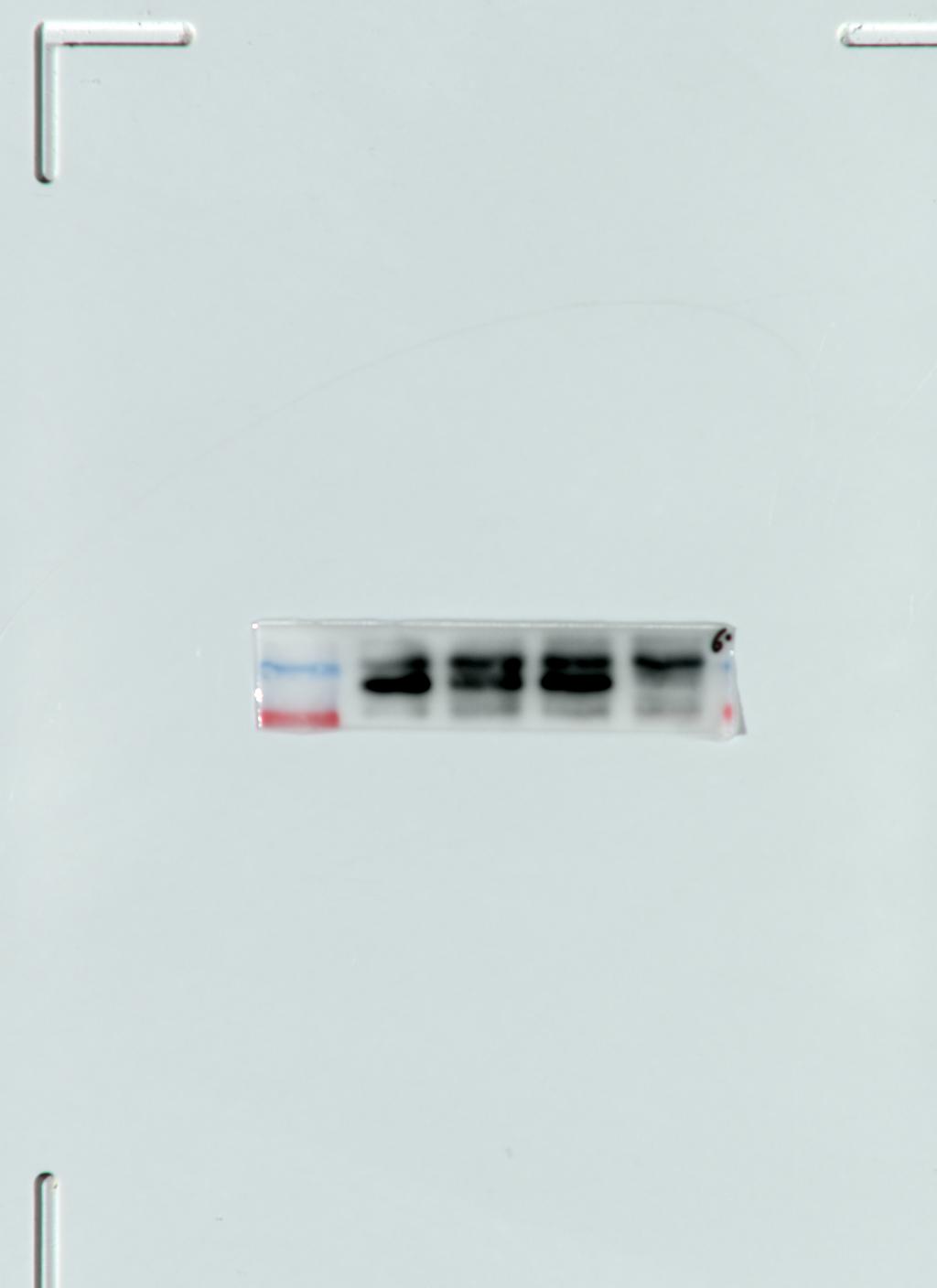

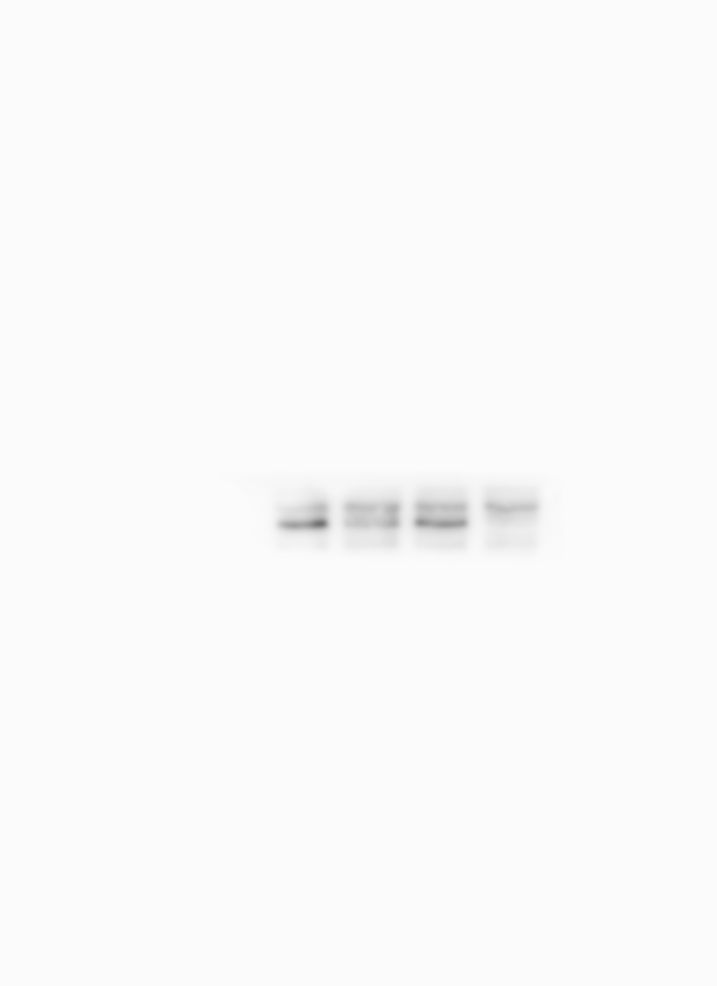


2020-8-27


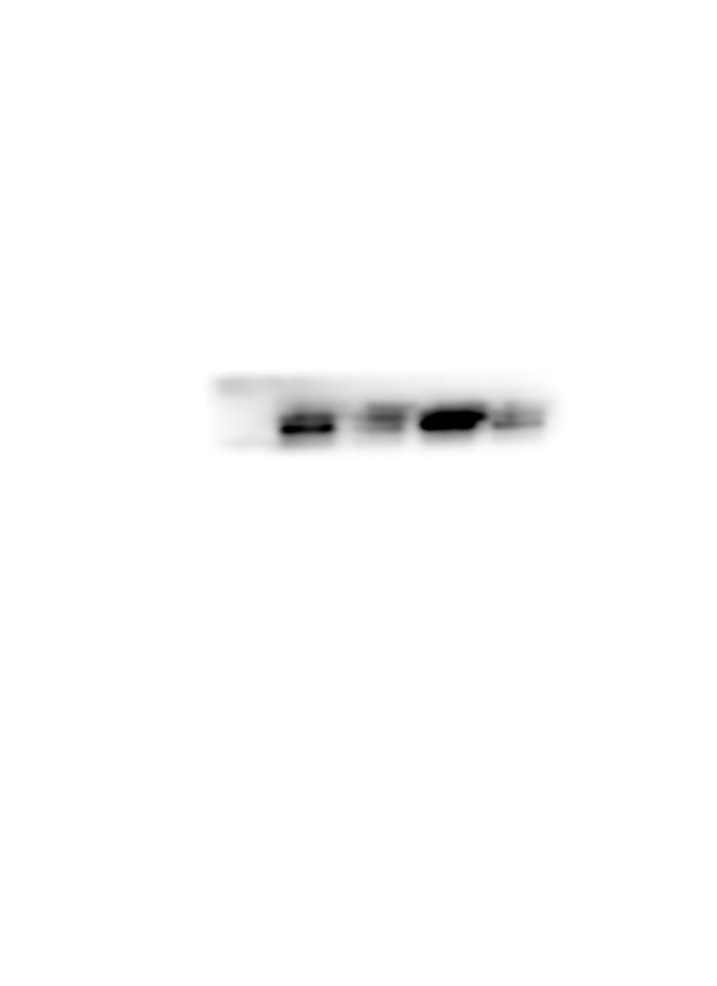

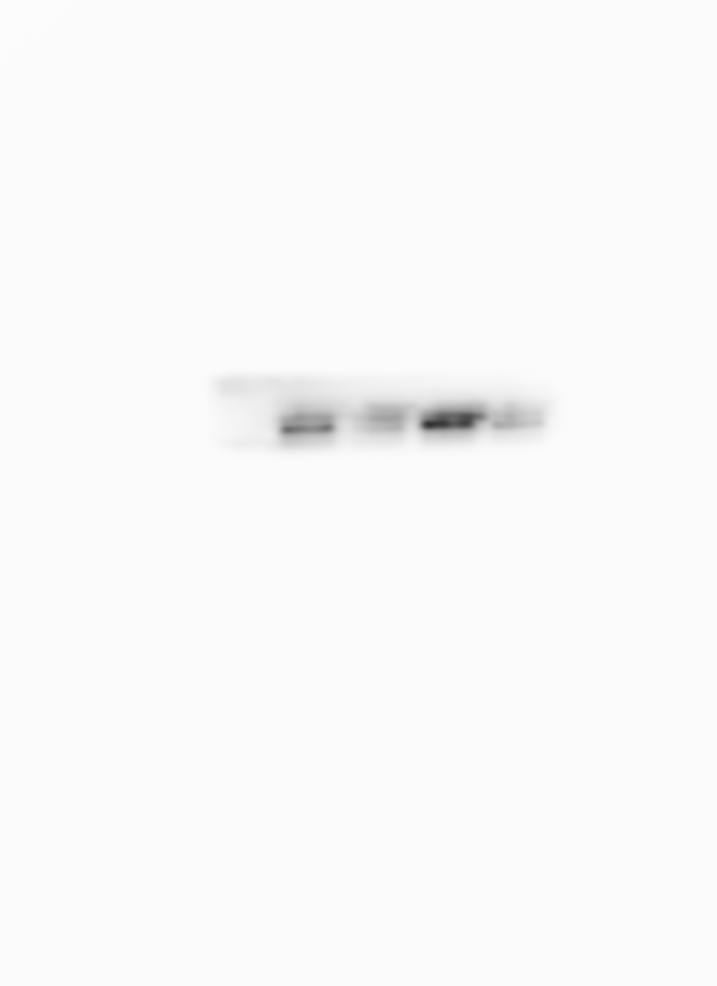

Supplement: Supplementary file 5 — Supplementary Information 5. [file 41598_2022_22617_MOESM5_ESM.docx]

2020-8-18


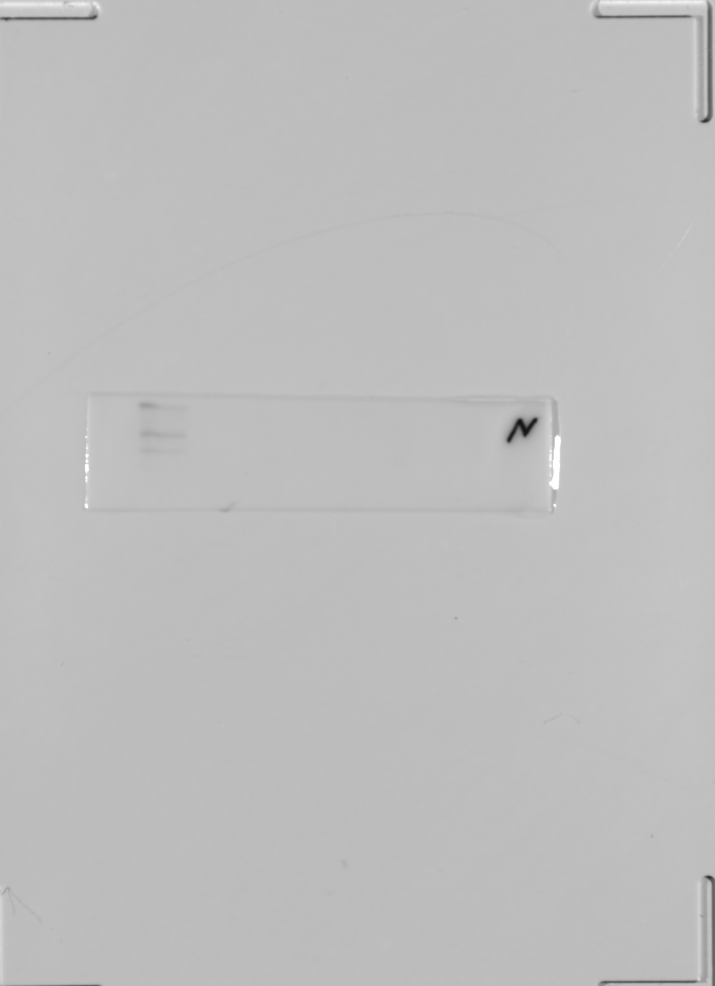

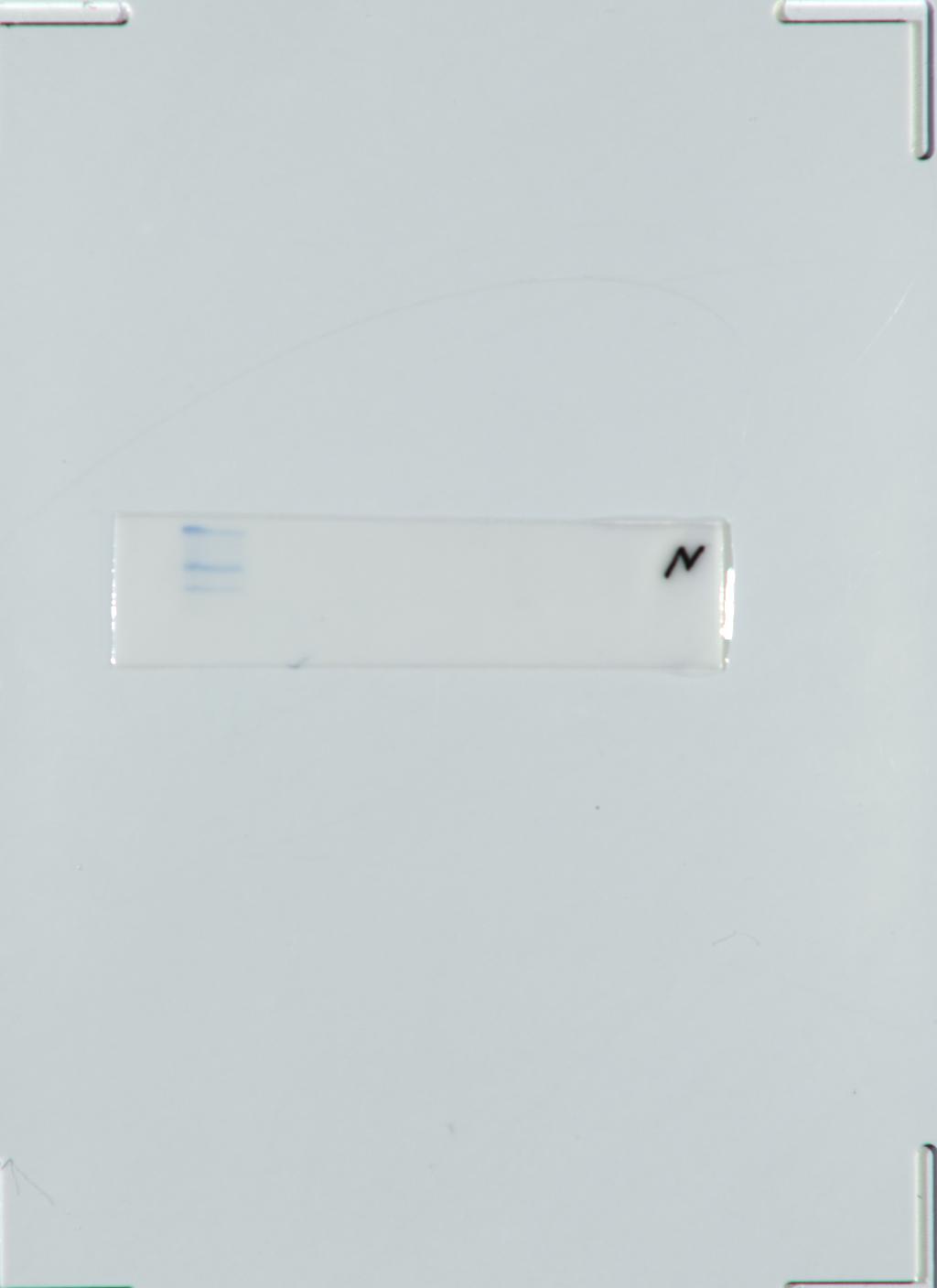

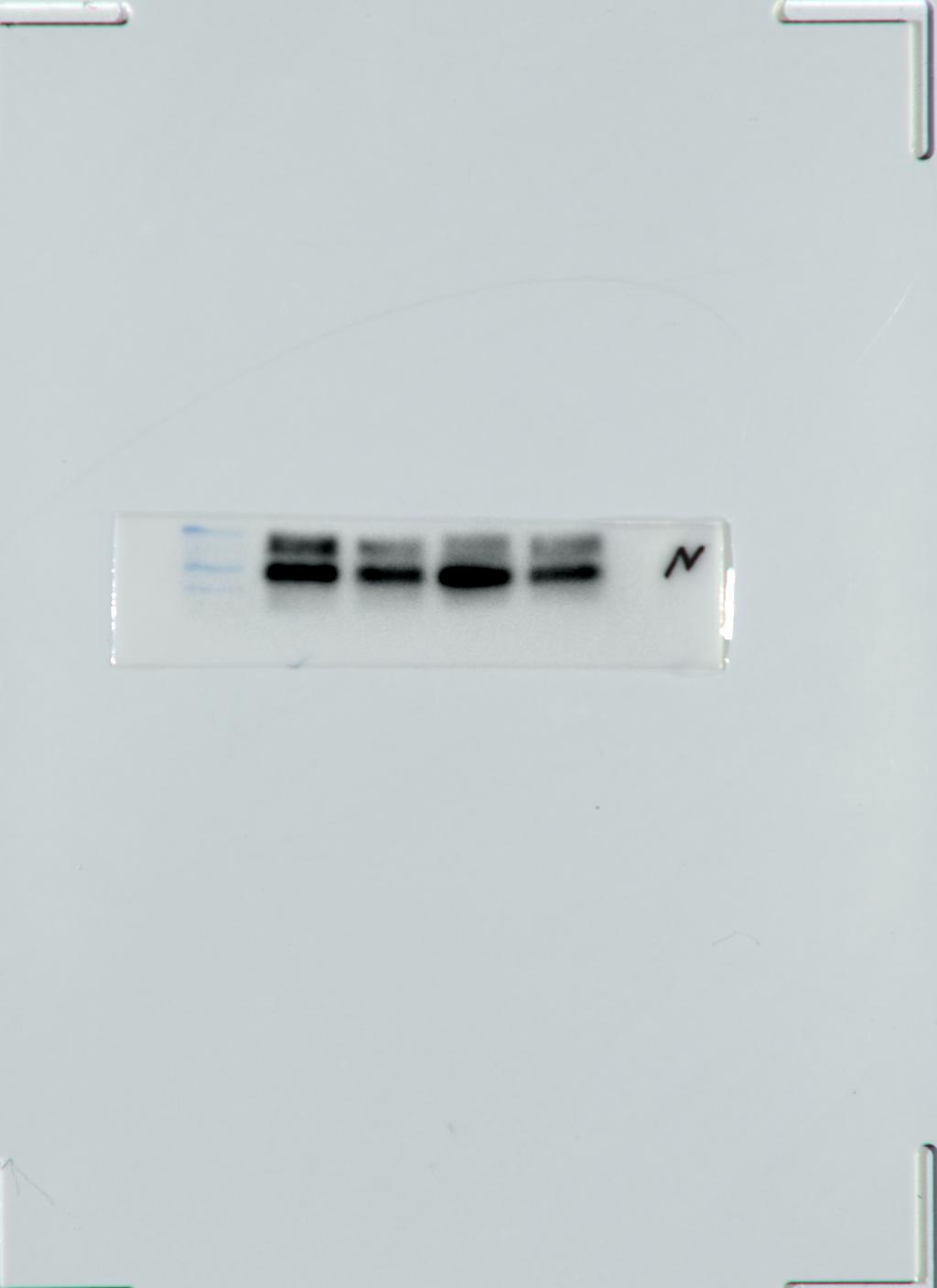

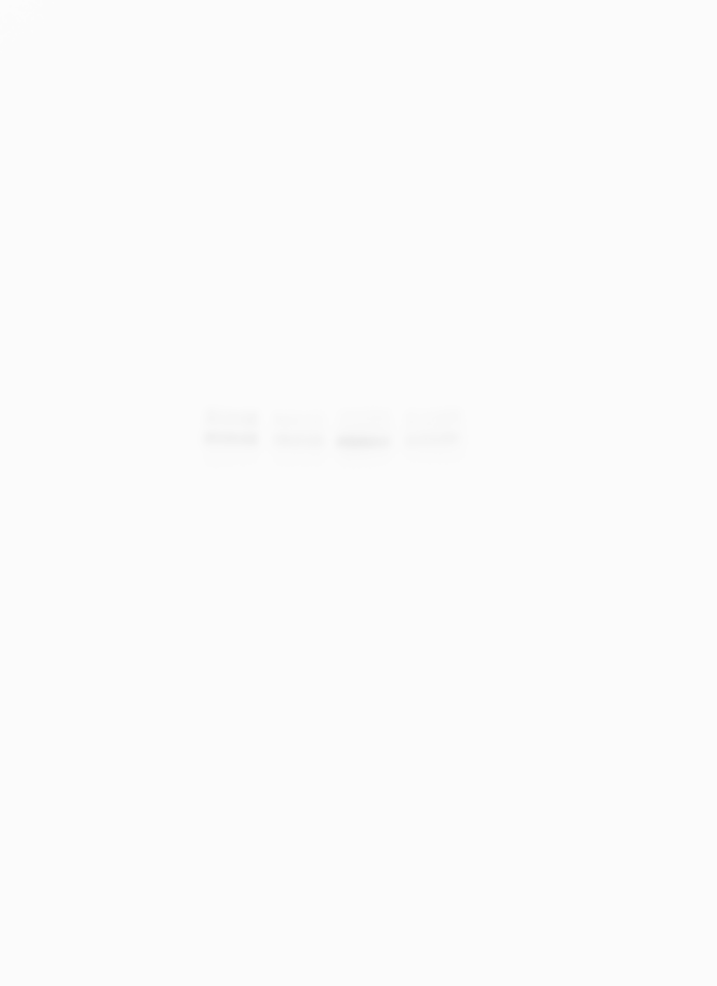


2020-8-21


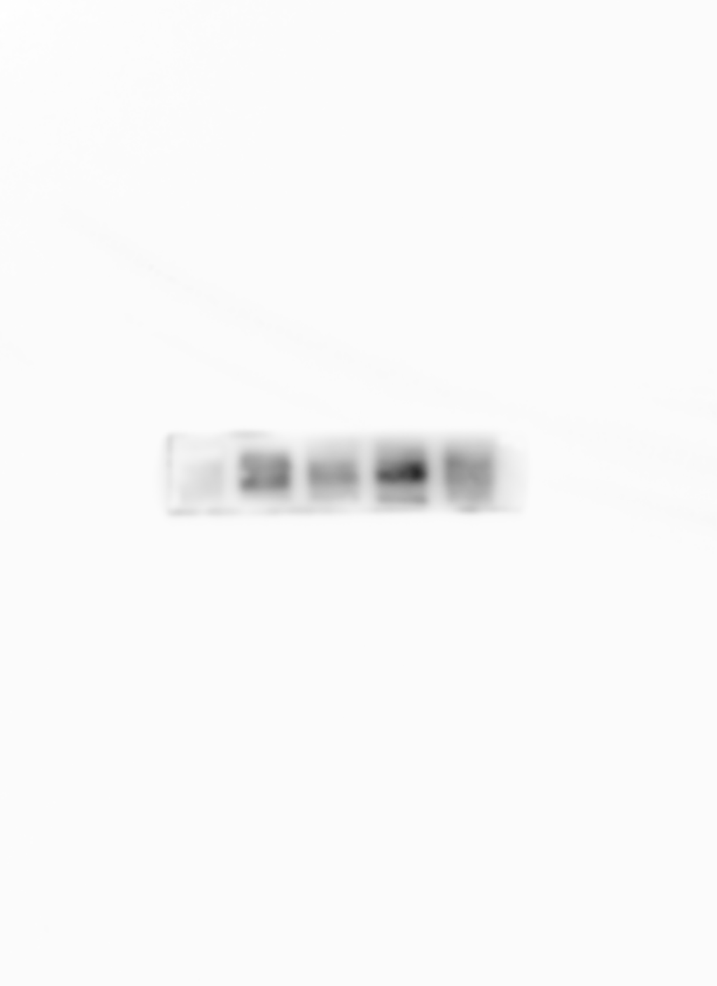

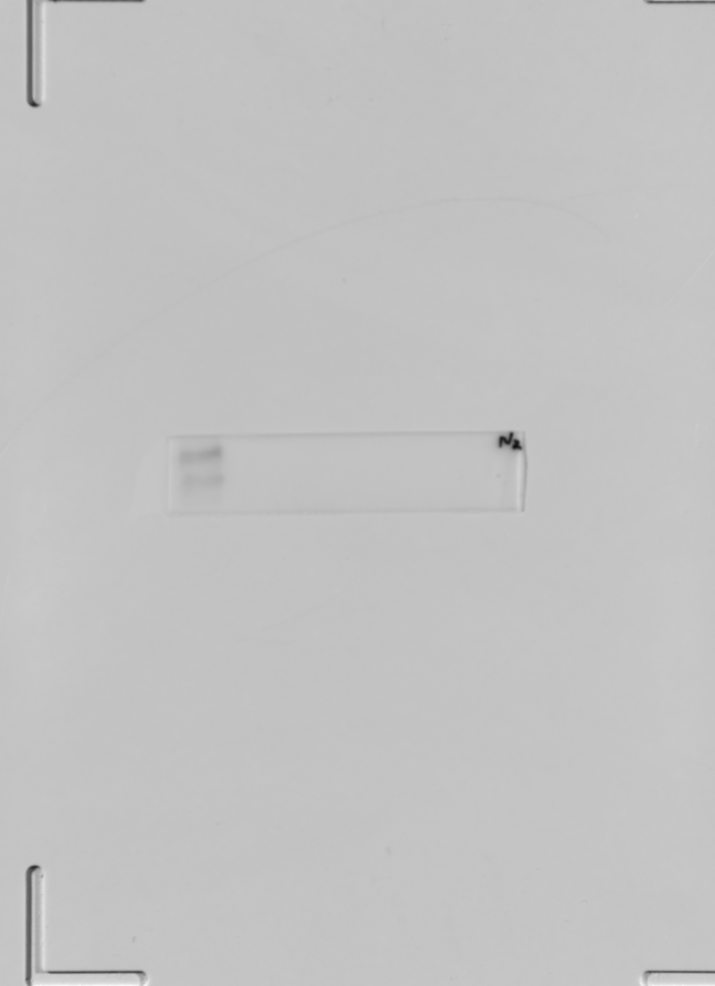

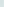

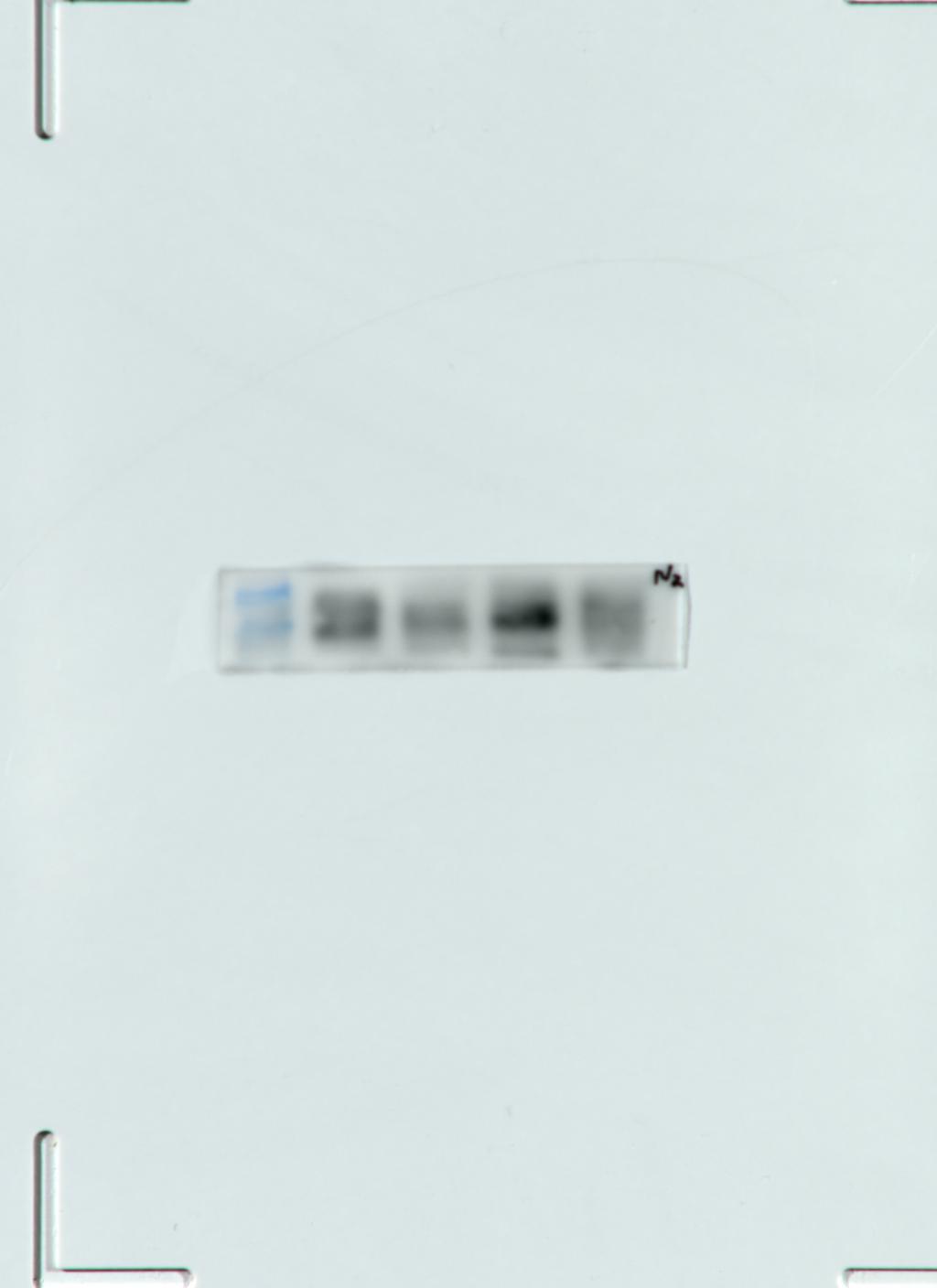


2020-8-27


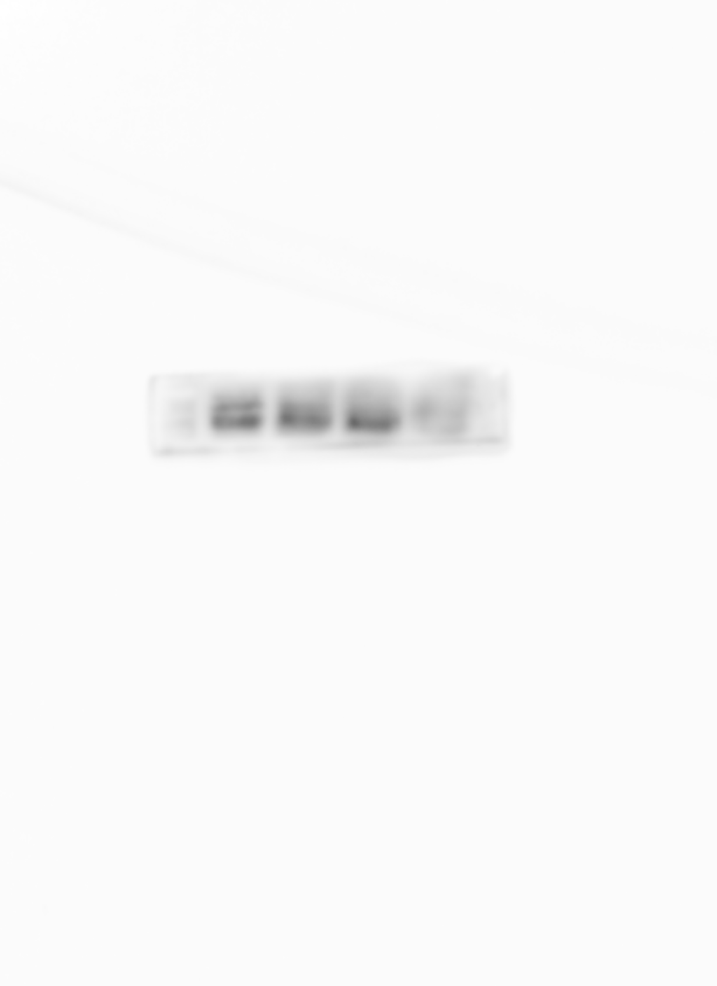

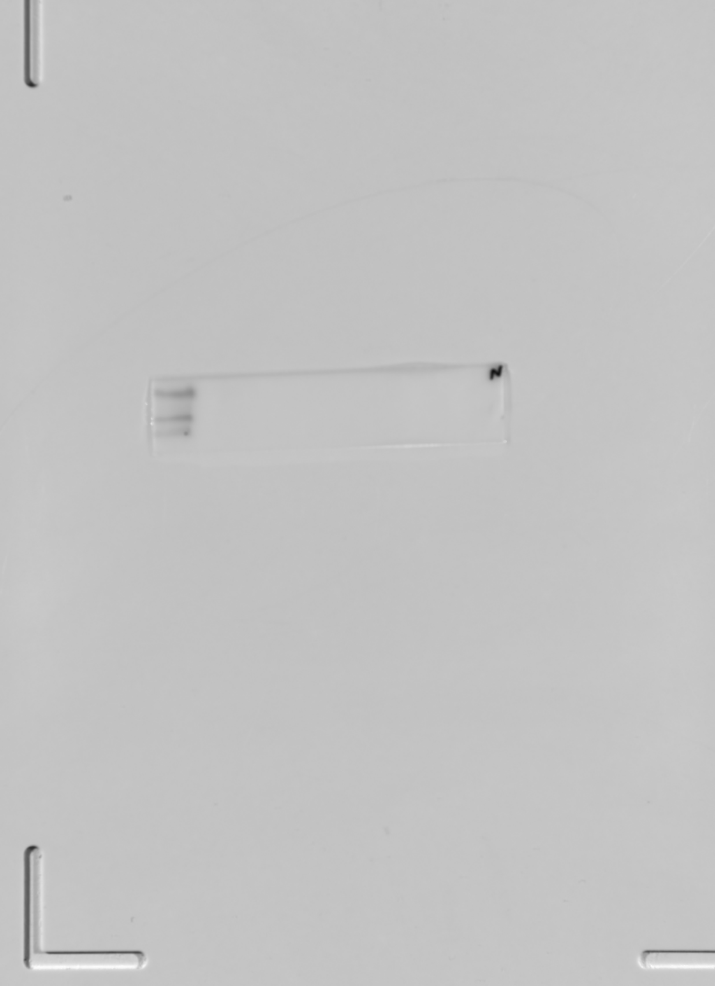

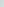

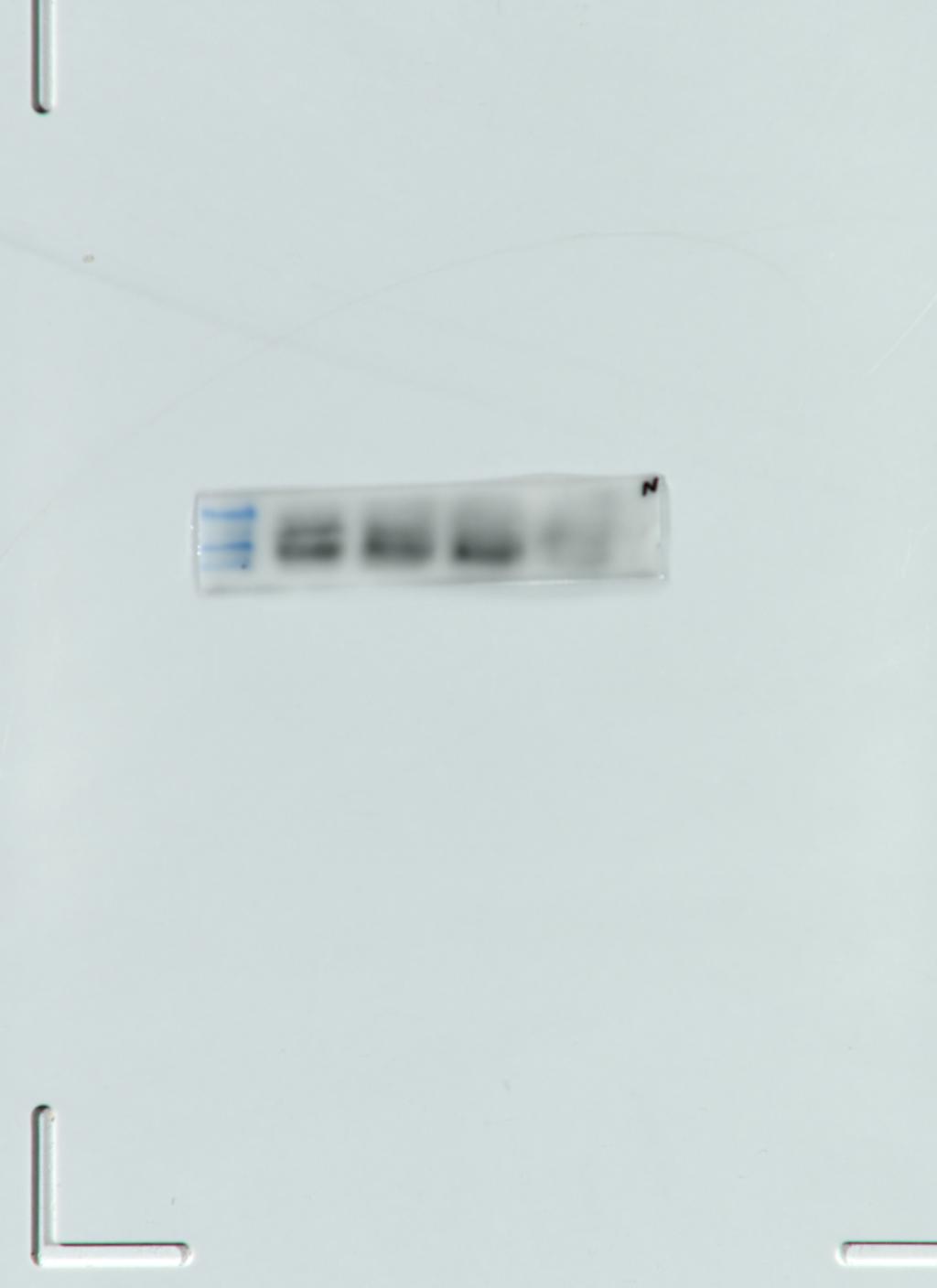

Supplement: Supplementary file 6 — Supplementary Information 6. [file 41598_2022_22617_MOESM6_ESM.docx]

2020-8-18


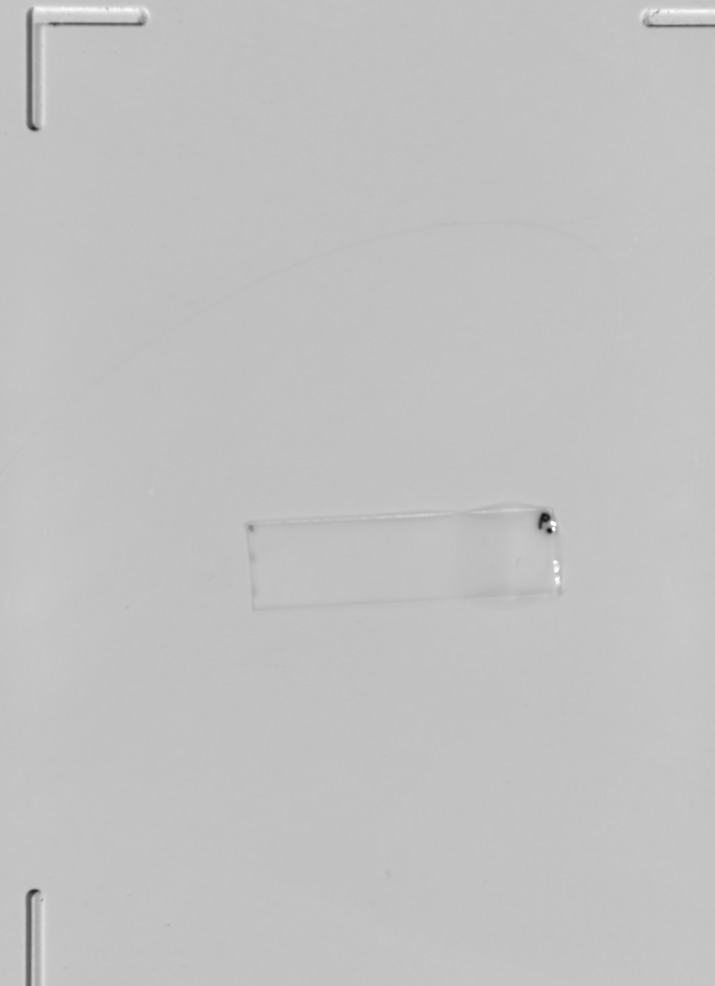

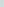

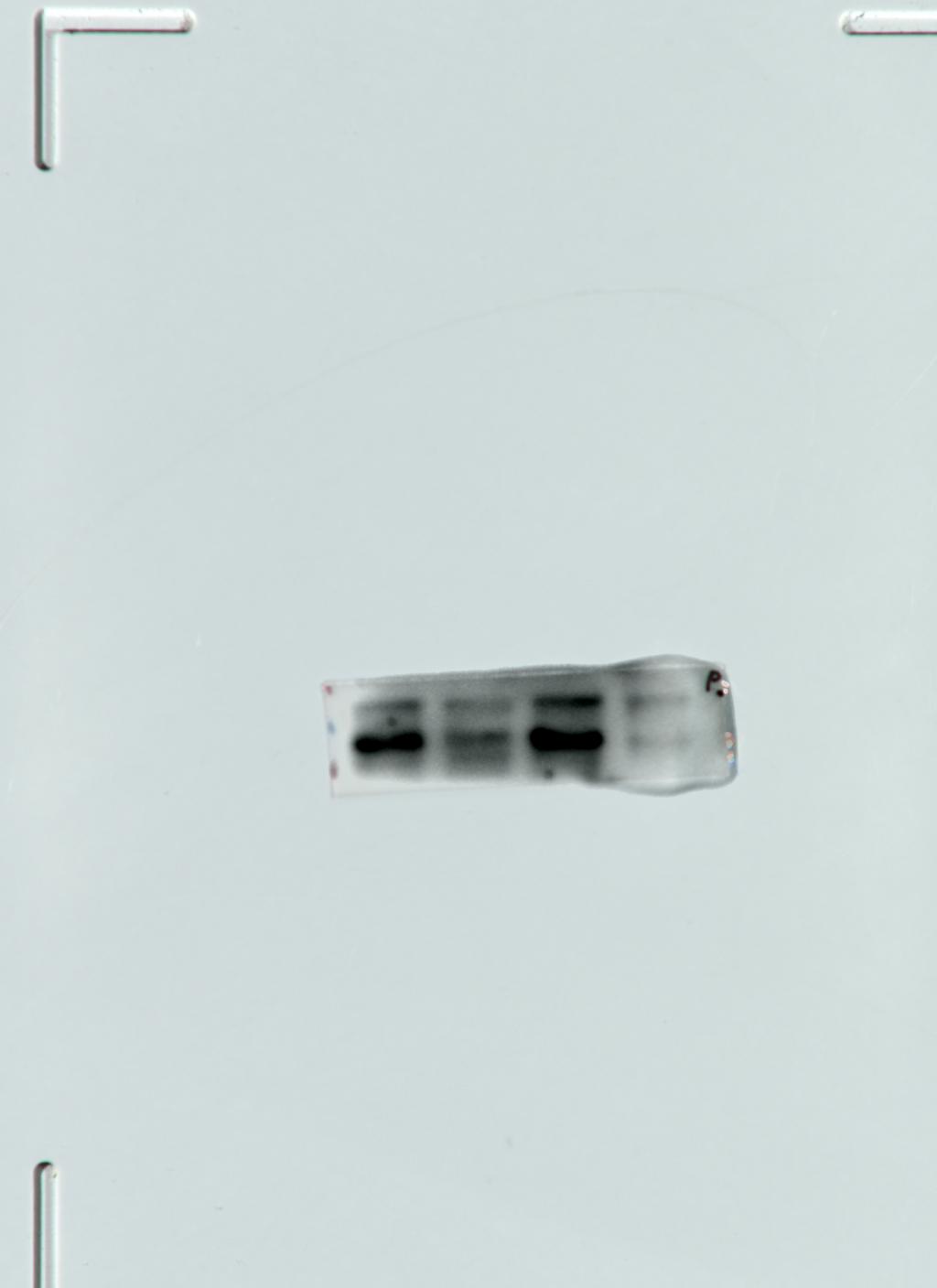

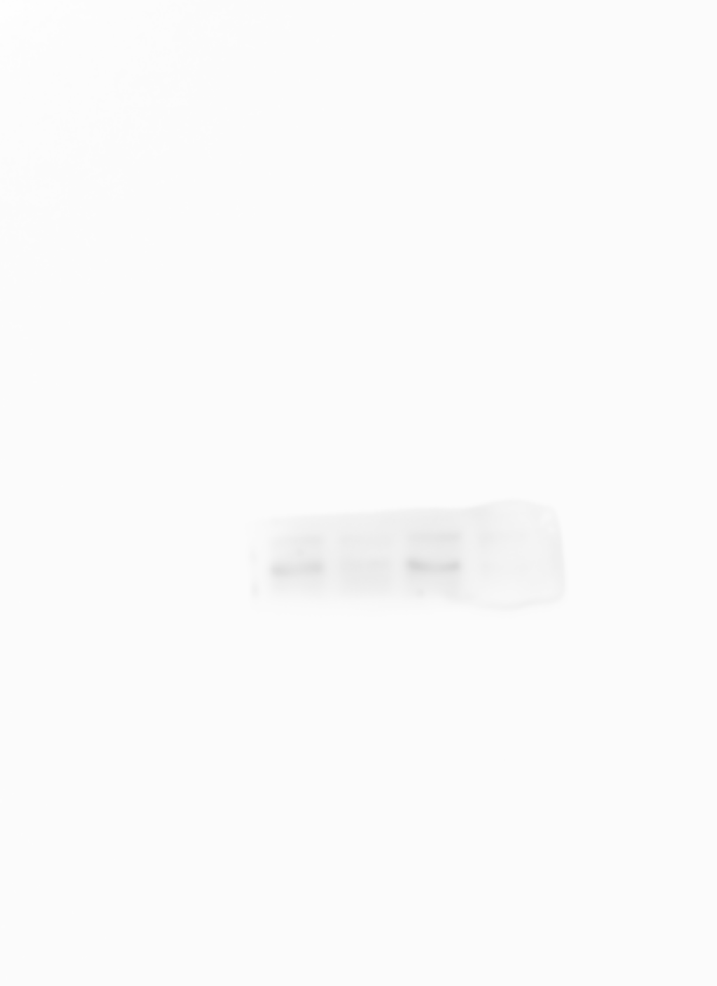

Supplement: Supplementary file 7 — Supplementary Information 7. [file 41598_2022_22617_MOESM7_ESM.docx]
